# Patients’ and caregivers’ experiences of familial and social support in resource-poor settings: A systematically constructed review and meta-synthesis

**DOI:** 10.1177/26323524251349840

**Published:** 2025-06-27

**Authors:** Yakubu Salifu, Emmanuel Ekpor, Jonathan Bayuo, Samuel Akyirem, Kennedy Nkhoma

**Affiliations:** 1 Division of Health Research, International Observatory on End of Life Care (IOELC), Faculty of Health and Medicine, Lancaster University, UK; 2 School of Psychology, Deakin University, Melbourne, VIC, Australia; 3 School of Nursing, The Hong Kong Polytechnic University, Kowloon, Hong Kong; 4 Yale School of Nursing, Yale University, West Haven, CT, USA; 5 Cicely Saunders Institute, King’s College London, UK

**Keywords:** familial support, life-limiting conditions, meta-synthesis, palliative care, resource-poor settings, social support

## Abstract

**Background:**

Familial and social support for patients with life-limiting conditions is crucial, especially in resource-poor settings. However, limited knowledge exists about patients’ and caregivers’ experiences within these informal networks in such contexts.

**Aims:**

This systematic review aimed to (i) synthesise the experiences of patients and caregivers regarding familial and social support in resource-poor settings, and (ii) understand the challenges they face in order to provide evidence for more compassionate, culturally congruent palliative care.

**Design:**

Systematic review and meta-synthesis registered on PROSPERO (CRD42023486219).

**Methods:**

We searched CINAHL, MEDLINE, PsycINFO, and Scopus using keywords such as “familial and social support” and “chronic debilitating conditions” in low- and middle-income countries. Only English-language qualitative studies exploring familial and social support were included. Thomas and Harden’s approach was used for data synthesis, and the Joanna Briggs Institute’s critical appraisal checklist was used to assess the studies’ quality.

**Results:**

We report our findings using the Enhancing Transparency in Reporting the Synthesis of Qualitative Research (ENTREQ) guidelines. Following screening, 39 studies were retained from 9157 search results. Five key themes emerged: (1) Bearing the weight of financial strain; (2) Psychosocial support as a “lifeline” for care; (3) Hands-on help and guidance; (4) Cultural and social obligations; and (5) Developing a “thick skin” and having faith as a coping mechanism. The findings show that caregiving in the context of life-limiting illnesses is influenced by financial burdens, emotional challenges, and cultural obligations, with caregivers depending on spiritual and social networks. However, resource availability is inconsistent, underscoring the need for tailored interventions.

**Conclusion:**

Culturally specific palliative care strategies are necessary to ease caregiver burdens, improve resource distribution, and support the well-being of patients and caregivers in resource-poor settings.

## Plain language summary

Understanding Caregiving Challenges and Support Needs in Low-Resource Settings

Families and social networks play a key role in caring for those with life-limiting conditions, especially in areas with limited healthcare. However, there is little understanding of the challenges faced by patients and caregivers relying on informal support. This review explored their experiences to improve culturally and socially relevant palliative care. A systematic search of academic databases identified 39 studies from an initial 9,157 results, focusing on family and community support in low- and middle-income countries. The review followed established qualitative research guidelines and assessed the studies’ quality. Five key themes emerged: First, financial strain is a significant burden, with caregivers struggling with costs and loss of income. Second, emotional and psychological support from family and community helps patients and caregivers cope with stress. Third, caregivers provide hands-on care, often without professional training. Fourth, caregiving is shaped by cultural expectations and family obligations. Lastly, faith and resilience help caregivers manage challenges, with many relying on spirituality. The study shows that while family and social networks are crucial, caregivers often feel overwhelmed due to financial hardship, emotional stress, and lack of resources. Spiritual and community support help, but gaps in healthcare services worsen the burden. Addressing these issues requires culturally sensitive palliative care policies and better resource distribution.

## Introduction

Familial and social support for patients with palliative care needs contributes significantly to their well-being. Capturing the needs of families and caregivers is a critical part of palliative care.^1^ However, the quality of healthcare and the support available to patients and their caregivers can vary significantly depending on the resources available in each setting.^2^ While challenges exist across all settings, they can be more pronounced in low- and middle-income countries (LMICs) due to systemic issues such as inadequate healthcare infrastructure, financial constraints, and limited access to trained healthcare providers. In resource-poor settings, individuals confronting illnesses often encounter unique obstacles in accessing adequate care and support. In this study, resource-poor settings are defined as environments—usually low- and middle-income or underserved countries—with limited access to essential healthcare resources, including medical infrastructure, trained personnel, medications, and funding.^3^ These challenges are characterised by limited financial resources, underdeveloped healthcare infrastructure, and disparities in healthcare services,^4^ creating a complex landscape for patients and their caregivers. In such challenging environments where access to quality healthcare services can be elusive, the significance of family and social support cannot be overstated.

Familial support encompasses the assistance provided by immediate family members and close relatives.^5^ This form of support can manifest in various ways, including instrumental support (e.g., shouldering the financial burden of medical expenses), therapeutic alliance (e.g., offering emotional support during difficult times) and other forms.^6^ Indeed, familial support can serve as a lifeline for patients and caregivers, bridging gaps left by inadequacies in the formal healthcare system in resource-poor settings. In LMICs, familial networks are often strong, with relatives more willing to provide care; however, this willingness is frequently hindered by a lack of financial and material resources, limiting their ability to offer consistent support. When familial support is strained or insufficient, social support can serve as a crucial alternative.^7^ Social support extends beyond the immediate family to include friends, neighbours, community groups, and formal support structures such as non-governmental organisations and charitable foundations.

Notwithstanding the critical role of familial and social support, individuals in resource-poor settings may not consistently receive the requisite support from these networks. Economic constraints in resource-poor settings can limit families’ ability to provide financial support for healthcare expenses, medications, or other necessities.^8^ Additionally, while familial networks may be willing to provide care, the stigma surrounding chronic illnesses—such as HIV, prostate cancer, and diabetes—can weaken the support system. Cultural beliefs that associate chronic conditions with impending death or “wasting resources” can further discourage families from providing adequate care.^9–12^ It is evident that familial and social support in resource-poor settings is dynamic, and deeply rooted in the economic, cultural, and social fabric of these environments. As patients and caregivers in resource-poor settings strain to meet basic needs, understanding the intricacies of their experiences becomes pivotal. Yet, there is a dearth of reviews exploring patients and caregivers’ experiences of support in the context of areas with limited healthcare and professional support. Available reviews have either focused on specific aspects of care, such as pain management^13^ or nutritional support^14^; particular care settings, such as emergency departments^15^ or for specific disease, for example, stroke.^16^ Venables’ study^17^ explored caregivers’ and healthcare professionals’ experience in India, but a single qualitative study is insufficient to provide evidence for care.

Consequently, this study seeks to fill this gap by providing a comprehensive synthesis of qualitative research from various resource-poor settings, offering evidence for more compassionate care that resonates with the real-life experiences of patients and caregivers facing resource constraints.

## Aim

The aim of this knowledge synthesis was to:

(i) synthesise the experiences of patients and caregivers regarding familial and social support in resource-poor settings; and
(ii) understand the challenges they face, in order to provide evidence for more compassionate, culturally congruent palliative care.

The systematic review was designed to address the following research questions:

1. How do patients and caregivers in resource-poor settings experience and interpret familial and social support in the context of managing health conditions?
2. What are the common challenges and facilitators related to familial and social support as experienced by patients and caregivers in resource-poor settings, and how do these influence health outcomes?

## Methods

### Study design

A meta-synthesis—a method for systematically reviewing and integrating qualitative research data from various studies to create a new interpretation of a research field—was conducted to consolidate evidence on patients’ and caregivers’ experiences of familial and social support in resource-poor settings.^18,19^ This approach facilitates the development of novel insights and concepts derived from constituent data. It allows for a deeper understanding of the contextual dimensions of findings across multiple studies, offering a comprehensive perspective on complex issues such as familial and social support in palliative care across different cultural and resource-poor settings.^20^

### Protocol registration

The study was developed and prospectively registered on PROSPERO (CRD42023486219).

### Search strategy

A systematic search for relevant studies was conducted across multiple databases, including CINAHL, MEDLINE, PsycINFO, and Scopus. Additionally, we reviewed the reference lists of retrieved articles to identify any further pertinent studies. Our search strategy incorporated key terms related to “familial and social support,” specific clinical conditions (e.g. cancer, diabetes, cardiovascular diseases, stroke, dementia, HIV, and COVID-19), and all LMICs as classified by the World Bank. To refine our search towards qualitative studies, we included terms specific to qualitative research methodologies and applied controlled vocabulary alongside relevant keywords. The “explode” function in MEDLINE and PsycINFO was used to capture all relevant subcategories, while Boolean operators (“AND,” “OR”) were strategically applied to optimise results.

Our search was initially conducted in November 2023 and was updated in March 2025 to ensure the inclusion of the most recent evidence. We limited our search to articles published from January 2000 to provide a comprehensive yet contemporary synthesis of the literature. The search strategy was refined with input from an academic reference librarian, whose expertise assisted in optimising the search strings. A full list of search terms and strategy details is provided in the Supplemental Materials.

### Eligibility criteria

Articles were included in this review if they had family caregivers, with or without patients, as study participants and:

(1) Were primary qualitative studies of any design.
(2) Focused on adult patients living with chronic or life-threatening/life-limiting illnesses such as cancer, diabetes, cardiovascular diseases, stroke, organ failure, dementia, HIV/AIDS, or COVID-19, along with their family caregivers.
(3) Were conducted in LMICs, based on the World Bank’s classification.^21^
(4) Specifically discussed the experiences of patients and/or caregivers in receiving and providing support in the context of illness.
(5) Were published in English. Only studies with direct quotes related to familial and social support were included. Participants were primary caregivers, consistent with the definition of a caregiver as “unpaid, informal providers of one or more physical, social, practical and emotional tasks. In terms of their relationship to the patient, they may be a friend, partner, ex-partner, sibling, parent, child or other blood or non-blood relative.”^22^

The exclusion criteria were review articles, conference abstracts, posters, dissertations, and other grey literature, any other language other than English, conducted before 2000. Additionally, studies involving conditions other than specified above were excluded. We also excluded studies focusing on paid caregivers such as nurses.

### Screening

Articles retrieved from the electronic search were imported into *EndNote 20* (Clarivate Analytics, Philadelphia, PA, USA) to remove any duplicates. The remaining articles were then uploaded onto *Rayyan* (Rayyan Systems Inc., Doha, Qatar), a collaborative systematic review management platform, for screening. Data screening followed the PRISMA statement.^23^ This involved an initial screening of study titles and abstracts. Subsequently, the full texts of the selected articles were carefully assessed to determine whether they met the inclusion criteria for the review. Screening and article selection were conducted independently by two reviewers (Y.S. and E.E.). Disagreements at any stage were resolved through discussion with a third reviewer (J.B.).

### Data extraction

A standardised data abstraction form in Excel format was employed to systematically capture pertinent information from each study. The extracted information included the author(s), name(s), publication year, study location, study aim, specific qualitative design, and data collection strategies. Additionally, data on the conditions and participants studied, and findings on familial and social support were extracted. The data extraction process was performed independently by two reviewers (E.E., Y.S.) and was cross-checked by a third reviewer (K.N.) to ensure accuracy and consistency. Any inadvertent discrepancies between the reviewers were resolved through discussion and consensus.

### Critical appraisal

Two reviewers (Y.S. and E.E.) independently appraised the quality of the studies using the Joanna Briggs Institute 10-item standardised critical appraisal checklist.^24^ A study is rated high quality (8–10), medium quality (5–7), or weak quality (1–4). Disagreements were resolved through discussion until consensus was achieved.

All included studies were evaluated as high or medium quality, with each receiving at least 7 “yes” ratings out of 10 on the appraisal checklist (see Table 1). Twenty-four studies were rated high quality, and 14 were rated medium quality; therefore, no article was excluded based on appraisal results. Most methodological flaws were related to:

(1) incongruity between the stated philosophical perspective and research methodology;
(2) insufficient cultural and theoretical positioning of the researcher; and
(3) lack of clarity on the influence of the researcher on the research, and vice versa.

**Table 1.** Quality assessment of included studies.

Author (year)	Quality assessment scores for each question										Overall score
	1	2	3	4	5	6	7	8	9	10	
Adam and Koranteng (2020)	✓	✓	✓	✓	✓	✗	—	✓	✓	✓	8
Adejoh et al. (2021)	—	✓	✓	✓	✓	✗	—	✓	✓	✓	7
Adejoh et al. (2024)	—	✓	✓	✓	✓	✗	—	✓	✓	✓	7
Alqaissi and Dickerson (2010)	✓	✓	✓	✓	✓	✗	—	✓	—	✓	7
Bahrami et al. (2014)	—	✓	✓	✓	✓	✗	—	✓	✓	✓	7
Banchani et al. (2020)	—	✓	✓	✓	✓	✗	—	✓	✓	✓	7
Biney et al. (2024)	—	✓	✓	✓	✓	—	—	✓	✓	✓	7
Binka et al. (2019)	✓	✓	✓	✓	✓	✗	—	✓	✓	✓	8
Brown et al. (2022)	—	✓	✓	✓	✓	✗	—	✓	✓	✓	7
Duodu et al. (2024)	✓	✓	✓	✓	✓	✗	—	✓	✓	✓	8
Hamid et al. (2021)	✓	✓	✓	✓	✓	✗	—	✓	✗	✓	8
Hendricks-Lalla and Pretorius (2020)	—	✓	✓	✓	✓	✗	✓	✓	✓	✓	8
Hesamzade et al. (2017)	—	✓	✓	✓	✓	✗	✓	✓	✓	✓	8
Hobenu and Naab (2023)	—	✓	✓	✓	✓	✗	—	✓	✓	✓	7
Jabeen et al. (2024)	✓	✓	✓	✓	✓	✓	✓	✓	✓	✓	10
Knight and Schatz (2022)	✓	✓	✓	✓	✓	✗	—	✓	✓	✓	8
Kusi et al. (2020)	✓	✓	✓	✓	✓	✓	✓	✓	✓	✓	10
Lelaka et al. (2022)	✓	✓	✓	✓	✓	✗	—	✓	✓	✓	8
Mbozi et al. (2023)	✓	✓	✓	✓	✓	✗	✓	✓	✓	✓	9
Mbozi et al. (2023)	—	✓	✓	✓	✓	✗	✓	✓	✓	✓	8
Mlaba et al. (2021)	✓	✓	✓	✓	✓	✗	—	✓	✓	✓	8
Mohammadian et al. (2023)	—	✓	✓	✓	✓	✗	✓	✓	✓	✓	8
Mokhtari et al. (2022)	✓	✓	✓	✓	✓	✗	—	✓	✓	✓	8
Moyer et al. (2014)	✓	✓	✓	—	✓	✗	—	✓	✓	✓	7
Mphasha et al. (2022)	✓	✓	✓	✓	✓	✗	—	✓	✓	✓	8
Musyimi et al. (2024)	—	✓	✓	✓	✓	✓	✓	✓	—	✓	8
Mwendwa et al. (2021)	✓	✓	✓	✓	✓	✗	—	✓	✓	✓	8
Najjuka et al. (2023)	✓	✓	✓	✓	✓	✗	✓	✓	✓	✓	9
Nankinga et al. (2020)	—	✓	✓	✓	✓	✗	—	✓	✓	✓	7
Nguyen et al. (2021)	—	✓	✓	✓	✓	✗	—	✓	✓	✓	7
Ninnoni and Owoo (2023)	✓	✓	✓	✓	✓	✗	—	✓	✓	✓	8
Nwakasi et al. (2023)	—	✓	✓	✓	✓	✗	—	✓	✓	✓	7
Sadeghi-Mahalli et al. (2024)	—	✓	✓	✓	✓	✗	✗	✓	✓	✓	7
Salifu et al. (2020)	✓	✓	✓	✓	✓	✓	✓	✓	✓	✓	10
Shahrbabaki et al. (2016)	—	✓	✓	✓	✓	✗	—	✓	✓	✓	7
Sheikhpourkhani et al. (2018)	✓	✓	✓	✓	✓	✗	—	✓	✓	✓	8
Tsedze et al. (2025)	—	✓	✓	✓	✓	—	✓	✓	✓	✓	8
Widyastuti et al. (2023)	✓	✓	✓	✓	✓	✗	✓	✓	✓	✓	9
Zeilani et al. (2022)	✓	✓	✓	✓	✓	✓	✓	✓	✗	✓	9

Critical appraisal questions: (1) Is there congruity between the stated philosophical perspective and the research methodology? (2) Is there congruity between the research methodology and the research question or objectives? (3) Is there congruity between the research methodology and the methods used to collect data? (4) Is there congruity between the research methodology and the representation and analysis of data? (5) Is there congruity between the research methodology and the interpretation of results? (6) Is there a statement locating the researcher culturally or theoretically? (7) Is the influence of the researcher on the research, and vice-versa, addressed? (8) Are participants, and their voices, adequately represented? (9) Is the research ethical according to current criteria or, for recent studies, and is there evidence of Research Ethics Committee approval by an appropriate body? (10) Do the conclusions drawn in the research report flow from the analysis, or interpretation, of the data?

✓: Yes;—: unclear; ✗: no.

## Results

### Study selection results

**Figure 1.**
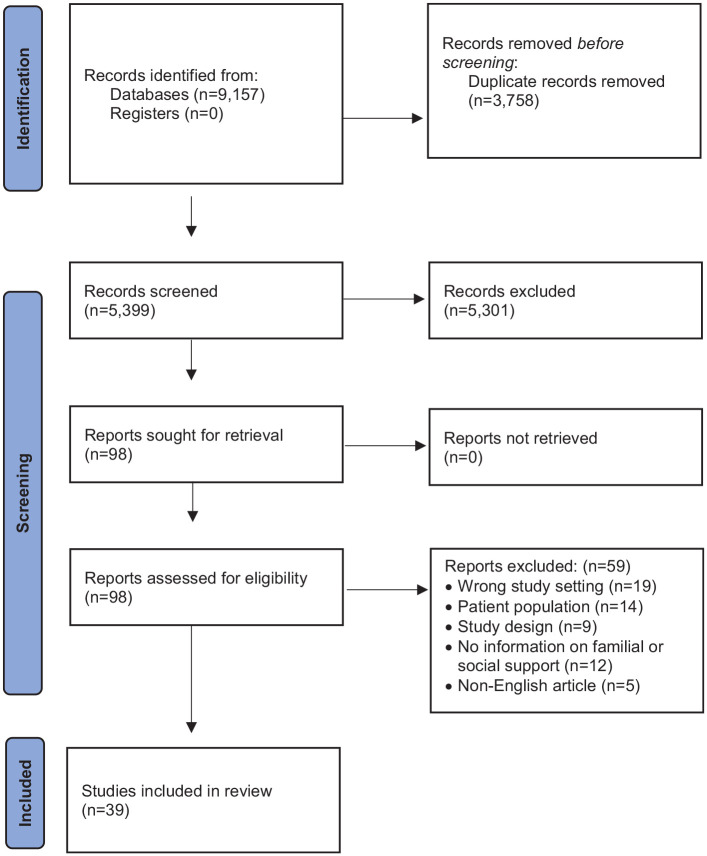
PRISMA flow chart.

The systematic search yielded 9157 records, including 8446 from our initial search. Duplicates of 3758 were removed (Figure 1). The remaining 5399 articles were screened in two stages: first, by assessing their titles and abstracts, and subsequently through full-text examination of potentially relevant studies (*n* = 98). Articles were excluded during full-text screening based on our predefined eligibility criteria, including non-alignment of study setting (*n* = 19), patient population (*n* = 14), study design (*n* = 9), focus on familial and social support (*n* = 12), and language of publication (*n* = 5).

In total, 39 articles published between 2010 and 2025 were included in this review (see Table 2). One of these was a cross-country study involving Nigeria, Uganda, and Zimbabwe.^25^ The studies were conducted across 13 countries—7 from Africa and 6 from Asia. Ghana had the highest representation with 10 studies, followed by Iran with 7. Data were predominantly collected through face-to-face interviews, except in two studies conducted online via telephone or Zoom.^26,27^ Cancer was the most common condition (*n* = 20), followed by dementia (*n* = 8), HIV (*n* = 5), and other conditions (*n* = 6). This study adhered to the Enhancing Transparency in Reporting the Synthesis of Qualitative Research (ENTREQ) guidelines to ensure a rigorous, transparent, and comprehensive synthesis of qualitative evidence.^28^ The ENTREQ guidelines provide a 21-item checklist designed to improve the clarity, completeness, and transparency of reporting in qualitative evidence syntheses, thereby enhancing the trustworthiness and usability of findings.

**Table 2.** Characteristics of included studies.

Author (year)	Aim	Country	Study design	Data collection/site	Condition	Participants
Adam and Koranteng (2020)	To assess the availability, accessibility, and impact of social support on treatment for breast cancer patients at Komfo Anokye Teaching Hospital, Ashanti Region in Ghana	Ghana	Phenomenological study	In-depth interview Face-to-face	Cancer (breast)	Women with breast cancer
Adejoh et al. (2021)	To understand the role, impact, and support of informal caregivers of patients with advanced cancer when interacting with palliative care services in Nigeria, Uganda, and Zimbabwe	Nigeria, Uganda, and Zimbabwe	Multi-country cross-sectional qualitative study	Individual semi-structured interviews Face-to-face	Cancer	Informal caregivers of patients with advanced cancer
Adejoh et al. (2024)	To explore the role of social capital in breast cancer management among women living with breast cancer	Nigeria	Qualitative study	In-depth interviews Face-to-face	Cancer (breast)	Women with breast cancer
Alqaissi and Dickerson (2010)	To explore common meanings of social support as experienced by Jordanian women with breast cancer	Jordan	Qualitative phenomenological study	Semi-structured individual interviews Face-to-face	Cancer (breast)	Women with breast cancer
Bahrami et al. (2014)	To explore the Iranian family caregivers’ burden of caregiving for patients with heart failure	Iran	Descriptive exploratory qualitative study	Semi-structured interviews Face-to-face	Heart failure	Family caregivers of the heart failure patients
Banchani et al. (2020)	To examine how social support assists in the management of NCDs—specifically hypertension, diabetes, and stroke—in Ghana	Ghana	Qualitative study	Semi-structured interviews Face-to-face	Non-communicable disease including hypertension, diabetes, and stroke	Persons with hypertension, diabetes, and stroke
Biney et al. (2024)	To identify the challenges and coping strategies developed by family caregivers to cope with the care of the terminally ill person	Ghana	Exploratory descriptive qualitative study	Semi-structured interview Face-to-face	Cancer	Family caregivers of terminally ill patients
Binka et al. (2019)	To explore male knowledge and support during cervical cancer screening and treatment for their cervical cancer patient partners in a rural setting in Ghana	Ghana	Qualitative study	In-depth interviews and focus group discussions Face-to-face	Cancer (cervical)	Cervical cancer patients, male partners of patients, other married men
Brown et al. (2022)	To explore familial support in integrated treatment with antiretroviral therapy and medications for opioid use disorder	Vietnam	Qualitative study	Semi-structured interviews Face-to-face	HIV	Persons with HIV and their family members
Duodu et al. (2024)	To explore family caregivers’ experiences on coping in dementia care	Ghana	Descriptive phenomenological study	Semi-structured interview Face-to-face	Dementia	Family caregivers of persons with dementia
Hamid et al. (2021)	To investigate the experiences of Kashmiri women with breast cancer regarding social support	India	Qualitative phenomenological study	In-depth interview Face-to-face	Cancer (breast)	Women with breast cancer
Hendricks-Lalla and Pretorius (2020)	To explore the experiences of male familial caregivers of persons with Alzheimer’s disease from low socio-economic status using the ecological systems theory perspective	South Africa	Qualitative study	In-depth semi-structured interviews Face-to-face	Dementia (Alzheimer’s disease)	Males family caregivers of persons with Alzheimer’s disease
Hesamzadeh et al. (2017)	To explore and describe family caregivers’ experiences about the strategies to handle ADL dependency of elderly patient with stroke in the Iranian context	Iran	Qualitative study	Semi-structured in-depth interview Face-to-face	Stroke	Family caregivers of persons with stroke
Hobenu and Naab (2023)	To explore the social experiences of women diagnosed with advanced cervical cancer in Ghana	Ghana	Qualitative explorative descriptive design	Semi-structured interview Face-to-face	Cancer (cervical)	Women with breast cancer
Jabeen et al. (2024)	To explore the experiences of family caregivers of advanced breast cancer patients	Pakistan	Qualitative study	In-depth interviews Face-to-face	Cancer (breast)	Family caregivers of patients with advanced breast cancer
Knight and Schatz (2022)	To explore social support for improved art adherence and retention in care among older people living with HIV in urban South Africa	South Africa	Descriptive qualitative study design	Semi-structured interviews Face-to-face	HIV	HIV-positive adults over 50 years old
Kusi et al. (2020)	To explore and describe the caregiving motivations and experiences among family caregivers of patients living with advanced breast cancer	Ghana	Exploratory descriptive phenomenological study	Semi-structured qualitative interviews Face-to-face	Cancer (breast)	Family caregivers of persons with breast cancer
Lelaka et al. (2022)	To examine the psychosocial support provided for HIV serodiscordant couples both in healthcare settings and in the community	South Africa	Interpretative phenomenological analysis design	In-depth interviews Face-to-face	HIV	HIV serodiscordant couples
Mbozi et al. (2023)	To explore the experiences and coping strategies of women caring for their husbands with cancer attending the cancer disease hospital	Zambia	Descriptive phenomenological study	In-depth interviews Face-to-face	Cancer	Female spouses caring for their husbands who have cancer
Mbozi et al. (2023)	To explore how African-centred values such as Ubuntu shape the social networks of people living with multimorbidity in South Africa and al-lows them to seek care, mobilise resources, and build resilience, even when living in constant precarity	South Africa	Qualitative study	Semi-structured interviews Face-to-face	HIV/NCD	People living with HIV/NCD multimorbidity
Mlaba et al. (2021)	To explore the social burden that families experience in providing care to their family members living with cancer	South Africa	Qualitative phenomenological study	Semi-structured interview Face-to-face	Cancer	Family caregivers of people with cancer
Mohammadian et al. (2023)	To explore the factors influencing support provision to the family caregivers of elderly patients with cancer	Iran	Descriptive qualitative study	In-depth semi-structured interviews Face-to-face	Cancer	Family caregivers of elderly patients with cancer, their family members, and healthcare providers
Mokhtari et al. (2022)	To explain the perceived experiences of women suffering from breast cancer towards social support	Iran	Qualitative study	Individual interviews Face-to-face	Cancer (breast)	Women with breast cancer
Moyer et al. (2014)	To examine the failure of family-based HIV-care relations and the ways that community-based organisations have become entangled in family relations	Kenya	Ethnographic study design	Participant observation, interviews, informal conversations, focus group discussions, and walking sessions	HIV	Support groups and community members
Mphasha et al. (2022)	To explore family support in diabetes management	South Africa	Phenomenological exploratory descriptive design	Semi-structured interviews Face-to-face	Diabetes	Persons with diabetes
Musyimi et al. (2024)	To explore the perceived drivers of care for PWD	Kenya	Qualitative study	Focus group discussion and individual interview Face-to-face	Dementia	Family caregivers of persons with dementia, healthcare providers, members of the general public
Mwendwa et al. (2021)	To explore the experiences of caring for a person living with dementia in Kenya	Kenya	Interpretative phenomenological study design	Semi-structured interview Face-to-face	Dementia	Families and paid caregivers of persons with dementia
Najjuka et al. (2023)	To explore the family caregivers/relatives’ experiences of caring for patients with advanced cancer	Uganda	Descriptive phenomenological study	In-depth interviews Face-to-face	Cancers	Family caregivers of patients with advanced cancer
Nankinga et al. (2020)	To establish the nature of informal support provided for PWD and its perceived usefulness in rural communities in Southwestern Uganda	Uganda	Qualitative descriptive design	In-depth interviews Face-to-face	Dementia	Family are givers of persons with dementia and community opinion leaders
Nguyen et al. (2021)	To examine the family caregiving experience in a semi-rural region outside of central Hanoi from the perspectives of family caregivers and other key informants	Vietnam	Descriptive qualitative study	Semi-structured interviews Face-to-face	Dementia	Family caregivers of PWD, healthcare providers, community leaders
Ninnoni and Owoo (2023)	To explore the psychosocial experiences of caring for the family caregiver of patients with prostate cancer	Ghana	Descriptive phenomenological study	In-depth semi-structured interviews Face-to-face	Cancer (prostate)	Family caregivers of prostate cancer patients
Nwakasi et al. (2023)	To investigate cancer survivors’ experiences with seeking care and support	Nigeria	Descriptive qualitative study design	Semi-structured interview Telephone	Cancer (breast)	Female breast cancer survivors
Sadeghi-Mahalli et al. (2024)	To identify the factors affecting the support of Iranian older spousal caregivers of people with Alzheimer’s disease	Iran	Qualitative content analysis	In-depth semi-structured interviews Face-to-face	Dementia (Alzheimer’s disease)	Family caregivers (older spouses) of persons with Alzheimer’s disease
Salifu et al. (2020)	To explore palliative and end-of-life care experiences of family caregivers and patients living at home in a resource-poor context in Ghana	Ghana	Thematic analysis	Face-to-face interview Dyad interview Focus group discussion	Prostate cancer	Men living with advanced prostate cancer Family caregivers Healthcare professionals
Shahrbabaki et al. (2016)	To explore the role of family support for patients with heart failure	Iran	Qualitative study	Semi-structured interviews Face-to-face	Heart failure	Persons with heart failure
Sheikhpourkhani et al. (2018)	To explore the perception of Iranian women with breast cancer regarding the role of social support in promoting their hope	Iran	Qualitative study	In-depth semi-structured interviews Face-to-face	Cancer (breast)	Persons with breast cancer
Tsedze et al. (2025)	To explore the experiences of patients’ knowledge of cardiovascular disease risk factors and coping strategies	Ghana	Descriptive phenomenological study	Semi-structured interview Face-to-face	Cardiovascular disease	Persons with cardiovascular disease
Widyastuti et al. (2023)	To explore the barriers and support for family caregivers in caring for older adults with dementia in Indonesia	Indonesia	Descriptive phenomenological study	Semi-structured individual interviews Online (telephone video call or zoom)	Dementia	Family caregivers of older adults with dementia
Zeilani et al. (2022)	To describe the experiences of family support from the perspectives of patients newly diagnosed with cancer	Jordan	Descriptive phenomenological study	In-depth interviews Face-to-face	Cancer	Newly diagnosed cancer patients

ADL: activities of daily living; NCD: non-communicable disease; PWD: people with dementia.

### Data synthesis

Thematic synthesis was employed to inductively extract descriptive and analytical themes from raw qualitative data, following Thomas and Harden’s approach.^29^ This method involved a step-by-step process that ensured thorough engagement with the data through free coding, aggregation of significant ideas into descriptive themes, and interpretation of these themes into core concepts.

In the first step, E.E. and Y.S. performed free coding on all quotes by assigning keywords to each data item, summarising their content and significance (see Supplemental Materials). These codes were then reviewed and cross-checked by two additional authors (J.B. and S.A.), with any discrepancies resolved through discussion and consensus during a team meeting.

In the second step, the codes were organised into a hierarchical coding tree to capture broader ideas and their subordinate categories. Descriptive themes were then developed to summarise these key ideas, representing potential barriers and facilitators of care. These themes were grounded in the raw data to preserve the participants’ voices.

Finally, in the third step, the descriptive themes were used to develop interpretive analytical themes, allowing new perspectives and concepts to emerge. This meta-synthesis was interpretive, adding new meaning to the findings of the primary studies rather than merely summarising them.

### Themes

This section presents the findings from the qualitative analysis of studies exploring the experiences of family caregivers and patients with life-limiting illnesses across different settings, with a focus on social support, caregiving burden, cultural obligations, and coping strategies. The analysis generated five main themes: (1) Bearing the weight of financial strain, (2) Psychosocial support as a “lifeline” for care, (3) Hands-on help and guidance, (4) Cultural and social obligations, and (5) Developing a thick skin and having faith as a coping mechanism. This is presented in Table 3.

**Table 3.** Themes—Familial and social support in palliative care.

Theme	Studies
Bearing the weight of financial strain	Adam and Koranteng (2020), Alqaissi and Dickerson (2010), Bahrami et al. (2014), Banchani et al. (2020), Brown et al. (2022), Hendricks-Lalla and Pretorius (2020), Hobenu and Naab (2023), Hamid et al. (2021), Jabeen et al. (2024), Kusi et al. (2020), Lelaka et al. (2022), Mbozi et al. (2023), Mohammadian et al. (2023), Mokhtari et al. (2022), Mphasha et al. (2022), Najjuka et al. (2023), Salifu et al. (2020), Shahrbabaki et al. (2016), Sheikhpourkhani et al. (2018), Widyastuti et al. (2023), Zeilani et al. (2022)
Psychosocial support as a “lifeline” for care	Adejoh et al. (2024), Hamid et al. (2021), Hendricks-Lalla and Pretorius (2020), Hesamzadeh et al. (2017), Hobenu and Naab (2023), Kusi et al. (2020), Lelaka et al. (2022), Mbozi et al. (2023), Mphasha et al. (2022), Mokhtari et al. (2022), Najjuka et al. (2023), Sadeghi-Mahalli et al. (2024), Salifu et al. (2020), Shahrbabaki et al. (2016), Sheikhpourkhani et al. (2018), Widyastuti et al. (2023), Zeilani et al. (2022)
Hands-on help and guidance	Adam and Koranteng (2020), Adejoh et al. (2024), Hamid et al. (2021), Hendricks-Lalla and Pretorius (2020), Hesamzadeh et al. (2017), Kusi et al. (2020), Mphasha et al. (2022), Musyimi et al. (2024), Nankinga et al. (2020), Najjuka et al. (2023), Sadeghi-Mahalli et al. (2024), Salifu et al. (2020), Widyastuti et al. (2023), Zeilani et al. (2022)
Cultural and social obligations	Adejoh et al. (2024), Hendricks-Lalla and Pretorius (2020), Jabeen et al. (2024), Kusi et al. (2020), Mohammadian et al. (2023), Mbozi et al. (2023), Mokhtari et al. (2022), Musyimi et al. (2024), Najjuka et al. (2023), Salifu et al. (2020), Shahrbabaki et al. (2016), Sheikhpourkhani et al. (2018)
Developing a thick skin and having faith	Adam and Koranteng (2020), Biney et al. (2024), Duodu et al. (2024), Hamid et al. (2021), Kusi et al. (2020), Mohammadian et al. (2023), Musyimi et al. (2024), Najjuka et al. (2023), Nwakasi et al. (2023), Tsedze et al. (2025), Zeilani et al. (2022)

#### Theme 1: Bearing the weight of financial strain

Financial and material support emerged as a critical factor in the caregiving experience.^23–38^ The studies highlighted the financial burden faced by caregivers, often exacerbated by the lack of comprehensive social welfare systems and health insurance. For instance, breast cancer patients in Ghana shared experiences of receiving sporadic financial assistance from family and friends, which eventually dwindled, leaving them with the sole responsibility for financing their care.^31^ Similarly, caregivers of patients with heart failure in Iran reported significant financial strain, with many resorting to loans to cover medical expenses, which led to feelings of despair about sustaining the care in the long term.^32^

. . . I had to take a loan for buying his heart machine . . . I feel that I cannot follow the treatment process in the future. (Bahrami et al.,^32^ p. 60)

In contrast, in some settings, extended family networks provided crucial—though inconsistent—financial support. In Indonesia, family members living outside the patient’s immediate vicinity contributed financially to the care of dementia patients, which was essential in managing ongoing medical costs.^26^ However, the dependency on such support networks often placed a strain on relationships, as not all family members were able or willing to contribute equally, leading to tension and feelings of inequity among caregivers.

The lack of financial resources not only impacted the quality of care but also led to compromises in other aspects of life, such as nutrition and housing. Patients in Ghana and Iran, for example, reported having to choose between spending on medical care and basic necessities like food and shelter, highlighting the precariousness of their situations.^31,32^

We are told to eat balanced diet every day, in the beginning I used to do as they told but now I don’t get it like so I’m forced to eat whatever food I get and my daughter has been helping with the house chores. (Adam & Koranteng,^31^ p. 9)

We are tired of spending so much money to receive the treatment . . . he has been hospitalized continuously . . . The government should pay more attention to us by giving more financial support and discount on the hospital’s costs. (Bahrami et al.,^32^ p. 11)

#### Theme 2: Psychosocial support as a “lifeline” for care

The emotional and psychological burden experienced by patients was a recurrent theme, with patients often experiencing significant distress, loneliness, and anxiety during their illness. Psychosocial support—such as encouraging words, emotional comfort, spiritual assurance, and constant companionship—was deemed significant for both patients and caregivers^21,25–29,33–35,37–39^ The studies revealed that emotional support—whether from family, friends, or religious communities—played a pivotal role in alleviating this burden.^30^ In Ghana, for example, caregivers helped breast cancer patients find solace in their faith, with many turning to prayer and religious counselling to cope with the emotional challenges of their condition.

Now she (patient) does not cry anymore because I always encourage her that God is on the throne and that He will heal her. I pray and share God’s words with her. These have really increased her faith in God. (Kusi et al.,^33^ p. 8)

Moreover, the emotional support provided by caregivers was critical in managing the psychological well-being of patients. Caregivers often took on the role of emotional anchors, offering comfort, encouragement, and hope to their loved ones. In some cases, this involved sharing positive messages, arranging pastoral visits, and engaging in spiritual practices that reinforced the patient’s faith and resilience.^33–36^ Nevertheless, the burden of maintaining a positive outlook often led to internal conflict, as caregivers had to mask their own fears and anxieties to protect the patient’s emotional state.

In some cultural contexts, the emotional support extended beyond the immediate family to include community members and religious leaders, who provided a sense of collective care and belonging. For instance, in Uganda, community visits and the involvement of church members were integral to the emotional support network for patients with advanced cancer, offering both the patient and their caregivers a broader support system to rely on during difficult times.

. . . I have family members, church members and friends who are helping me. Church members have come home, like three or four times. They come as a group, and they sit with us, whatever they have, they give it to us. Also, our friends who are sending us money. . . those with money come at home and give us what they have. (Najjuka et al.,^52^ p. 14)

Emotional and psychological support from family, caregivers, and religious communities was crucial in alleviating distress, highlighting the need for community-centred emotional care in palliative services.

#### Theme 3: Hands-on help and guidance

Practical and informational support played a critical role in caregiving, offering both tangible help and guidance.^21,23,25–27,29,35,39–41^ These support are needed to navigate complex health conditions and treatments.^37–40^ Practical support mostly involved assisting with daily activities including bathing, grooming, meal preparation, and feeding, especially when the care recipient was physically unable to manage on their own.^34,37,39,41^ Additionally, family caregivers assisted patients with tasks, such as administering medications and attending medical appointments.^26,42,43^ This form of support was contributed to the overall stability and well-being of the care recipient.^26^

Now, my father’s physical health is stable because he regularly saw a doctor. I had a brother-in-law who assisted in accompanying my father to medical appointments. (Widyastuti et al.,^26^ p. 193)

It’s mostly trying one thing or the other to see which one works best. We do “trial and error” most times honestly. We are on our own when we are at home. Healthcare is not my field of training; mine is in accounting, and I don’t know how to nurse big wounds. (Salifu et al.,^44^ p. 102)

Informational support, on the other hand, involved sharing relevant information, offering advice, and helping others understand their medical conditions or prescribed regimens. For instance, caregivers extended physiotherapist’s instructions into the home environment, ensuring continuity of care and empowering the patient to actively participate in their own rehabilitation.^37^ Caregivers also played an essential role in medication adherence by not only reminding the patient of their schedule but also explaining the rationale behind the prescriptions.^38^ Additionally, family members with first-hand experience of certain conditions, provided practical insights to patients, offering clarity on what to expect in the course of their illness.^39^ This form of support was essential for patients to make informed decisions about their health and feel reassured during their care journey.

My cousin is a breast cancer survivor. . . . She tells me what’s going to happen step by step. . .. She told me you have stage II, and yours is early so you will be fine . . .. (Alqaissi & Dickerson,^39^ p. 358)

Practical and informational support from family caregivers was essential for maintaining the stability and well-being of patients, ensuring continuity of care beyond the clinical setting. This support helped patients make informed decision and manage their disease. Furthermore, it enhanced their overall care experience and quality of life.

#### Theme 4: Cultural and social obligations

Cultural and social obligations significantly shaped the caregiving experience across different settings. In many cultures, caregiving was seen as a moral duty, deeply rooted in societal norms and expectations.^25,26,28,32–34,36,37,44,45^ In Ghana, for instance, the role of caregiving was often dictated by cultural norms, with family members, particularly women, feeling a strong sense of obligation to care for their sick relatives.^33^ This cultural expectation was not only a source of pride but also a heavy burden, as caregivers struggled to balance their duties with other personal and professional responsibilities.^27^

We want to show him love and support by giving back what he did for us when we were young and fragile. (Salifu et al.,^44^ p. 203)

She is my mother, my family . . . it is actually my socio-cultural responsibility to take care of her . . . That is the reason I am the one taking care of her. (Kusi et al.,^33^ p. 8)

Caregiving as a form of social obligation also emerged from the concept of reciprocity,^33,46,47^ where individuals feel a moral duty to “repay” the care and support they once received from others. This sense of obligation was driven by societal norms, cultural expectations, or personal values that promote the exchange of care within relationships, even beyond familial bonds. For example, one participant shared:

It is unusual for someone who is not your family member to take care of you when you are sick, but she (patient) did it passionately when I was admitted at the hospital. So now that she is sick, it is my turn to repay her for what she did for me. (Kusi et al.,^33^ p. 8)

Similarly, in other contexts such as Iran, caregiving for elderly patients with chronic conditions such as heart failure and stroke was perceived as a familial obligation, with caregivers expressing a sense of duty that stemmed from both cultural and religious beliefs.^37,38^ These obligations often meant that caregivers had little choice but to take on the role, even when it led to significant personal and financial sacrifices. The societal pressure to fulfil these roles was compounded by the lack of formal support systems, making caregiving an all-encompassing responsibility that left little room for caregivers to attend to their own needs.

In contrast, the studies from South Africa and Kenya highlighted the role of extended family and community networks in fulfilling caregiving obligations. The concept of Ubuntu, which emphasises communal care and support, was particularly evident in South Africa, where caregiving was often shared among family members and the broader community, thereby reducing the burden on any one individual.^43,46,48,49^ However, even in these contexts, the expectation to provide care could lead to feelings of guilt and inadequacy among caregivers, particularly when they were unable to meet the needs of their loved ones due to financial or logistical constraints.

. . . I had good friends that supported me and my neighbour, she would sleep over, to give me a break . . . My family like I said, my son moved in with me to help me, gave up his place to be here to support me as he could and then if she got really sick . . . her friends pick up the phone, they come flying down here take her to hospital. (Mlaba et al.,^48^ p. 5)

Cultural and social obligations strongly influence caregiving, caregivers often feeling a deep moral duty to provide care, rooted in societal norms and reciprocity. This sense of responsibility can lead to significant personal and financial sacrifices, highlighting the need for formal support systems in palliative care to alleviate the heavy burden on caregivers and improve overall care outcomes.

#### Theme 5: Developing a thick skin and having faith as coping mechanisms

Caregivers employed various coping mechanisms to manage the physical, emotional, and psychological demands of caregiving.^23,24,29,44,50^ The concept of “*thick skin*” refers to the emotional resilience and fortitude that caregivers develop over time to protect themselves from the stress and emotional strain associated with caregiving. This coping strategy enables them to endure challenging situations without being overwhelmed by them, helping them maintain a sense of stability. Religious and spiritual practices emerged as common strategies across many settings.^32–34,36–38^ In Ghana and Uganda, for example, caregivers frequently turned to prayer, religious rituals, and spiritual counselling as sources of strength and resilience.^33,51,52^ These practices not only provided emotional relief but also fostered a sense of hope and purpose, which was crucial in sustaining the caregivers’ commitment to their roles.^53,54^

In addition to spiritual coping mechanisms, social support networks played a critical role in helping caregivers manage their responsibilities. In many cases, extended family members, friends, and community groups provided practical assistance, such as helping with household chores or offering financial support, which alleviated some of the burdens faced by primary caregivers. In Indonesia, for instance, the collaborative efforts of family members in caring for dementia patients allowed caregivers to share the load, thereby reducing stress and preventing burnout.^26^

The primary family members consistently offer support, even if not all of them reside in Semarang. Those living outside the city provide financial assistance” “If I need to be away from home for three days, my sister takes over the responsibility of caring for our mother. (Widyastuti et al.,^26^ p. 7)

. . . it’s not comfortable doing that all by myself. I need to ensure that I direct the affairs relating to care to avoid confusion and all that [addressing different duties all at the same time]. The task is not as simple as that. (Salifu et al.,^44^ p. 101)

Furthermore, some caregivers developed personal coping strategies, such as time management, seeking professional help, or engaging in hobbies, to maintain their well-being. In South Africa, caregivers of persons with HIV/non-communicable diseases multimorbidity reported using a combination of structured daily routines and social activities to manage their stress and maintain a sense of normalcy in their lives.^43^ However, the effectiveness of these coping mechanisms varied depending on the availability of resources and the caregivers’ social and economic circumstances. Together, these components—“thick skin” (emotional resilience) and practical coping strategies (such as time management or seeking social support)—complement each other, allowing caregivers to navigate both emotional and practical challenges. The development of “thick skin” provides the emotional endurance necessary to cope with the stress of caregiving, while personal coping strategies enable caregivers to better manage their daily responsibilities and stress levels. These findings are discussed with the view of understanding and addressing the diverse needs of caregivers, and how palliative care can be improved for a better outcome for both patients and their families.

## Discussion

This study sought to explore the experiences of patients and caregivers in resource-poor settings, focusing on familial and social support in the context of various chronic health conditions. The five themes—bearing the weight of financial strain, psychosocial support as a “lifeline” for care, hands-on help and guidance, cultural and social obligations, and developing a thick skin with faith—highlight the multifaceted challenges faced by patients and caregivers in palliative care. Together, these themes underscore the necessity of a holistic approach to palliative care that addresses not only medical needs but also emotional, cultural, and practical aspects of caregiving. The findings of this study provide a nuanced understanding of the experiences of patients with life-limiting illnesses and their family caregivers, highlighting critical aspects of support, burden, and coping strategies across diverse settings.

While the challenges caregivers face in resource-poor settings are substantial,^44-47,55^ it is important to acknowledge that many of these challenges are not unique to LMICs. Caregivers across different settings, including higher-income countries (HICs), experience financial strain, emotional burdens, and a lack of adequate social support networks.^56–59^ These issues are exacerbated in LMICs due to limited resources, but they are nonetheless common across various cultural and socio-economic contexts. Future research could explore what strategies are being implemented in HICs to address these challenges and compare them to the solutions in LMICs. This could lead to a better understanding of both the similarities and differences in caregiving experiences across diverse settings, though it is important to note that this study is one of the first to specifically focus on the experiences of caregivers in resource-poor settings, an area that has often been overlooked in research.

The findings of this analysis underscore the complex and multifaceted nature of caregiving for patients with life-limiting illnesses across different cultural and socio-economic contexts. Financial and material support, while essential, is often insufficient and inconsistently provided, leaving caregivers to shoulder significant burdens. Though these challenges are felt more acutely in LMICs, they are also present in HICs, where caregiving can still lead to financial and emotional strain, though often with a different structural context. Emotional and psychological support, particularly through religious and spiritual practices, plays a critical role in helping both caregivers and patients cope with the challenges of illness. However, this support is often contingent on the availability of resources,^60^ which can vary greatly between LMICs and HICs. Cultural and social obligations heavily influence the caregiving experience, often dictating who takes on the role and how it is perceived within the community. Finally, the resilience of caregivers is bolstered by a combination of personal coping mechanisms and external support networks, though the effectiveness of these strategies is contingent on the availability of resources.

These findings highlight the need for more comprehensive support systems that address not only the financial and practical needs of caregivers but also their emotional and psychological well-being. Future research should focus on developing culturally appropriate interventions that leverage existing social structures, such as community and religious networks, to provide emotional and practical support, especially in resource-poor settings. However, it would also be important to investigate how these social structures are utilised in higher-income settings and explore how differences in these approaches can inform solutions in LMICs.

### Financial and material support

Financial and material support emerged as a significant theme, where social welfare systems are often inadequate. Previous studies reported that family caregivers of individuals at the end-of-life face substantial financial strain, contributing to a multidimensional burden.^61,62^ This precarious financial situation can force patients and caregivers to make difficult decisions, such as choosing between healthcare and basic necessities like food and housing, which ultimately compromises the quality of care and the well-being of both parties.^63^ These findings align with existing literature that emphasises the need for more robust financial support mechanisms for families,^64^ especially in resource-poor settings.^61,62,65^

### Emotional and psychological support

Emotional and psychological support was another key theme identified in the analysis. The studies revealed that emotional support from family, friends, and religious communities is crucial in alleviating the psychological burden experienced by both patients and caregivers. This aligns with research by Cowan,^66^ which found that community-based emotional support significantly improves the mental health outcomes of caregivers. However, the burden of maintaining a positive outlook often places additional emotional strain on caregivers, as they struggle to balance their own anxieties with the need to support the patient.^67–69^ The role of religious and spiritual support in coping with emotional stress was particularly prominent in settings like Ghana and Uganda, where faith-based practices provide a sense of hope and community.^52^ This finding is consistent with studies highlighting the role of spirituality in coping with chronic illness.^13,68,70,71^

### Hands-on help and guidance

Hands-on help and guidance underscores the vital role that practical and informational support plays in caregiving. Family caregivers not only assist with daily activities like bathing, grooming, and meal preparation but also provide crucial support in managing complex health conditions and treatments, such as administering medications and attending medical appointments.^44,72–75^ This hands-on help is fundamental to maintaining the care recipient’s stability and well-being, ensuring they can continue receiving proper care even when they are physically unable to manage on their own. Additionally, informational support, such as sharing medical advice, explaining treatment regimens, and providing emotional reassurance, empowers patients to take an active role in their own care.^44^ This form of support enhances patients’ understanding of their health conditions, fosters informed decision-making, and boosts their confidence in managing their illness and ensuring patients’ dignity.^76,77^

### Cultural and social obligations

The analysis underscored the significant impact of cultural and social obligations on caregiving, particularly the moral and filial duties that caregivers often feel due to societal norms. In many settings, caregiving is seen as an inherent responsibility, particularly for women, rooted in cultural and religious values.^1,44,49,73^ This sense of obligation, while a source of pride, can lead to significant personal, financial, and professional sacrifices, as caregivers are often left with little choice but to assume the role, even at great personal cost.^78^ The concepts of *reciprocity* and *filial piety* are deeply embedded in cultural norms, compelling caregivers to “repay” the care they once received, reinforcing mutual support within families and communities.^44,54,79^ This sense of duty not only reflects moral responsibility but also demonstrates profound respect and obedience, particularly towards the elderly.^80,81^ In contrast, in South Africa, the communal concept of Ubuntu helps distribute the caregiving burden more evenly across extended family and community members.^82^ These findings highlight the crucial role that cultural norms and social obligations play in caregiving, but also emphasise the limitations these systems can impose, especially in the absence of formal support structures.^83^ Further exploration of cultural and social obligations, particularly comparing LMIC and HIC settings, would offer valuable insights into how caregiving is structured differently across diverse cultural contexts, thereby addressing the unique challenges faced by caregivers in resource-poor environments. The findings illustrate the deep-rooted sense of duty, gratitude, and societal expectations that shape familial caregiving, where support for an ailing loved one is driven by emotional commitment, moral responsibility, and cultural values.^1 [Bibr bibr2-26323524251349840],2,3,10,44,83^

### Coping mechanisms and resilience

Coping mechanisms and resilience were found to be essential for managing the physical, emotional, and psychological demands of caregiving. Religious and spiritual practices, along with social support networks, emerged as critical strategies for maintaining caregiver well-being.^33,44^ These coping mechanisms, however, are often contingent on the availability of resources and the caregivers’ social and economic circumstances. For instance, caregivers in New Zealand and Uganda reported using structured daily routines and social activities were better able to manage their stress and maintain a sense of normalcy.^84,85^ This pattern of improved coping through routine and social engagement is consistent with studies suggesting that structured interventions can enhance caregiver resilience.^4,86^

In light of these findings, there are several recommendations for future research, practice, and policy. First, future studies should explore the development of culturally appropriate interventions that address the specific needs of caregivers, particularly in resource-poor settings. These interventions should leverage existing social structures, such as community and religious networks, to provide emotional and practical support. Second, policies should focus on establishing comprehensive support systems that include financial assistance, emotional counselling, and respite care for caregivers; and how care is genedered, with men’s contribution often overlooked or undervalued within familial caregiving contexts due to prevailing norms that associate care with women.^87^ This would help alleviate the burden on families and improve the quality of care for patients. Lastly, there is a need for more research into the cost-effectiveness of different models of palliative care, as this could inform resource allocation decisions and improve the sustainability of care provision. Incorporating the perspectives of patients and caregivers into the development of these policies and interventions is crucial to ensure that they are truly responsive to the needs of those they are designed to support.

## Strengths and limitations

We conducted a comprehensive search using numerous synonyms for key terms and included interdisciplinary, multinational co-authors to ensure diverse perspectives and cultural viewpoints informed the meta-analysis. This is one of the first meta-analyses to systematically synthesise patients’ and caregivers’ experiences of palliative care in LMICs, filling a significant gap in the literature. However, the evidence included in the review is limited by its reliance on qualitative data from resource-poor settings, which may affect generalisability, and by the limited exploration of cultural and socio-economic variations across regions, possibly missing important nuances. The review process is also limited by the exclusion of non-English publications, which may have led to the omission of important studies published in other languages and the underrepresentation of certain cultural contexts, potentially overlooking additional work in this emerging area.

In light of these findings, several recommendations emerge:

(i) *Culturally appropriate interventions*: Develop interventions tailored to the cultural, social, and economic contexts of LMICs, leveraging existing social structures such as community and religious networks to provide emotional and practical support.
(ii) *Comprehensive support systems*: Establish policies that offer financial assistance, emotional counselling, and respite care for caregivers, aiming to alleviate their burden and improve the quality of care for patients.
(iii) *Cost-effectiveness of care models*: There is a need for more research into the cost-effectiveness of different models of palliative care, as this could inform resource allocation decisions and improve the sustainability of care provision. Incorporating the perspectives of patients and caregivers into the development of these policies and interventions is crucial to ensure that they are truly responsive to the needs of those they are designed to support.
(iv) *COVID-19 lessons*: The pandemic highlighted the urgent need for adaptable, community-driven palliative care models. Expanding telehealth services, integrating remote emotional support systems, and improving access to essential medications are key takeaways that should inform future policy and practice.

## Conclusion

This study sheds light on the complex dynamics of familial and social support in resource-poor settings, emphasising the critical role these networks play in shaping health outcomes for patients and caregivers. The findings underscore the importance of addressing the economic, social, and cultural barriers that hinder support, while also leveraging community strengths to create more resilient support systems. Moving forward, it is essential to develop policies and interventions that not only enhance access to healthcare but also strengthen the social and familial networks that are vital to the well-being of individuals in resource-poor settings. The findings of this study are relevant and timely given the high value of community-based support and the pressing need to strengthen community capacity in palliative care.

## Supplemental Material

sj-docx-1-pcr-10.1177_26323524251349840 – Supplemental material for Patients’ and caregivers’ experiences of familial and social support in resource-poor settings: A systematically constructed review and meta-synthesis Supplemental material, sj-docx-1-pcr-10.1177_26323524251349840 for Patients’ and caregivers’ experiences of familial and social support in resource-poor settings: A systematically constructed review and meta-synthesis by Yakubu Salifu, Emmanuel Ekpor, Jonathan Bayuo, Samuel Akyirem and Kennedy Nkhoma in Palliative Care and Social Practice

sj-docx-2-pcr-10.1177_26323524251349840 – Supplemental material for Patients’ and caregivers’ experiences of familial and social support in resource-poor settings: A systematically constructed review and meta-synthesis Supplemental material, sj-docx-2-pcr-10.1177_26323524251349840 for Patients’ and caregivers’ experiences of familial and social support in resource-poor settings: A systematically constructed review and meta-synthesis by Yakubu Salifu, Emmanuel Ekpor, Jonathan Bayuo, Samuel Akyirem and Kennedy Nkhoma in Palliative Care and Social Practice
