# Supplementary material for: Patients’ and caregivers’ experiences of familial and social support in resource-poor settings: A systematically constructed review and meta-synthesis
Source: Palliat Care Soc Pract. 2025 Jun 27;19:26323524251349840. doi: 10.1177/26323524251349840 (PMC12205196; doi:10.1177/26323524251349840)
Supplement: sj-docx-1-pcr-10.1177_26323524251349840 – Supplemental material for Patients’ and caregivers’ experiences of familial and social support in resource-poor settings: A systematically constructed review and meta-synthesis [file sj-docx-1-pcr-10.1177_26323524251349840.docx]

Database search strategy

**CINAHL**

| Concept | Search strings |
| --- | --- |
| #1 | (MH "Support, Social+") or (MH "Family Support") or TI (support N1 (social or famil* or friend* or spouse* or partner* or sibling* or relation* informal or informational or tangible or peer or friend* or organization* or functional or instrumental or material)) or AB (support N1 (social or famil* or friend* or spouse* or partner* or sibling* or relation* informal or informational or tangible or peer or friend* or organization* or functional or instrumental or material)) |
| #2 | (MH "Diabetes Mellitus") or TI (“diabetes mellitus” or diabetes or “type 1 diabetes” or “type 2 diabetes”) or AB (“diabetes mellitus” or diabetes or “type 1 diabetes” or “type 2 diabetes”) |
| #3 | (MH "Human Immunodeficiency Virus") OR (MH "HIV Infections") or (MH "Acquired Immunodeficiency Syndrome") or TI (HIV or “Human Immunodeficiency Virus” or AIDS or “Acquired immunodeficiency syndrome”) or AB (HIV or “Human Immunodeficiency Virus” or AIDS or “Acquired immunodeficiency syndrome”) |
| #4 | (MH "COVID-19") or TI (COVID-19 or coronavirus or “coronavirus disease”) or AB (COVID-19 or coronavirus or “coronavirus disease”) |
| #5 | (MH "Neoplasms") or TI (neoplasm* or cancer* or tumor* or malignan*) or AB (neoplasm* or cancer* or tumor* or malignan*) |
| #6 | (MH "Stroke") or TI (stroke or “ischemic stroke” or “cerebrovascular accident”) or TI (hemorrhage N1 (cerebral or intracerebral or brain)) or AB (stroke or “ischemic stroke” or “cerebrovascular accident”) or AB (hemorrhage N1 (cerebral or intracerebral or brain)) |
| #7 | (MH "Cardiovascular Diseases") or TI (“cardiovascular disease” or “cardiac arrest” or “heart failure” or “myocardial infarction” or “myocarditis” or angina or arrythmia*) or AB (“cardiovascular disease” or “cardiac arrest” or “heart failure” or “myocardial infarction” or “myocarditis” or angina or arrythmia*) |
| #8 | (MH "Dementia") or (MH "Alzheimer's Disease") or TI (dementia or Alzheimer disease) or AB (dementia or Alzheimer disease) |
| #9 | #2 or #3 or #4 or #5 or #6 or #7 or #8 |
| #10 | TI ((Low$ or poor$) N1 (income or resource) N1 (setting or countr*)) or TI (resource$poor or resource$limited or resource$restricted or LIC or LMIC) or AB ((Low$ or poor$) N1 (income or resource) N1 (setting or countr*)) or AB (resource$poor or resource$limited or resource$restricted or LIC or LMIC) |
| #11 | TI (Afghanistan or Albania or Algeria or Angola or Argentina or Armenia or Azerbaijan or Bangladesh or Belarus or Belize or Benin or Bhutan or Bolivia or "Bosnia and Herzegovina" or Botswana or Brazil or Bulgaria or "Burkina Faso" or Burundi or "Cabo Verde" or Cambodia or Cameroon or "Central African Republic" or Chad or China or Colombia or Comoros or "Congo" or "Democratic Republic of Congo" or "Costa Rica" or "Cote d'Ivoire" or Cuba or Djibouti or Dominica or "Dominican Republic" or Ecuador or Egypt or "El Salvador" or "Equatorial Guinea" or Eritrea or Eswatini or Ethiopia or Fiji or Gabon or Gambia or Georgia or Ghana or Grenada or Guatemala or Guinea or Guinea-Bissau or Haiti or Honduras or India or Indonesia or Iran or Iraq or Jamaica or Jordan or Kazakhstan or Kenya or Kiribati or "Korea, Democratic People's Republic" or Kosovo or "Kyrgyz Republic" or "Lao PDR" or Lebanon or Lesotho or Liberia or Libya or Madagascar or Malawi or Malaysia or Maldives or Mali or "Marshall Islands" or Mauritania or Mauritius or Mexico or "Micronesia, Federated States" or Moldova or Mongolia or Montenegro or Morocco or Mozambique or Myanmar or Namibia or Nepal or Nicaragua or Niger or Nigeria or "North Macedonia" or Pakistan or Palau or "Papua New Guinea" or Paraguay or Peru or Philippines or "Russian Federation" or Rwanda or Samoa or "Sao Tome and Principe" or Senegal or Serbia or "Sierra Leone" or "Solomon Islands" or Somalia or "South Africa" or "South Sudan" or "Sri Lanka" or "St. Lucia" or "St. Vincent and the Grenadines" or Sudan or Suriname or "Syrian Arab Republic" or Tajikistan or Tanzania or Thailand or Timor-Leste or Togo or Tonga or Tunisia or Turkey or Turkmenistan or Tuvalu or Uganda or Ukraine or Uzbekistan or Vanuatu or "Viet Nam" or "West Bank and Gaza" or "Yemen, Republic" or Zambia or Zimbabwe) or AB (Afghanistan or Albania or Algeria or Angola or Argentina or Armenia or Azerbaijan or Bangladesh or Belarus or Belize or Benin or Bhutan or Bolivia or "Bosnia and Herzegovina" or Botswana or Brazil or Bulgaria or "Burkina Faso" or Burundi or "Cabo Verde" or Cambodia or Cameroon or "Central African Republic" or Chad or China or Colombia or Comoros or "Congo" or "Democratic Republic of Congo" or "Costa Rica" or "Cote d'Ivoire" or Cuba or Djibouti or Dominica or "Dominican Republic" or Ecuador or Egypt or "El Salvador" or "Equatorial Guinea" or Eritrea or Eswatini or Ethiopia or Fiji or Gabon or Gambia or Georgia or Ghana or Grenada or Guatemala or Guinea or Guinea-Bissau or Haiti or Honduras or India or Indonesia or Iran or Iraq or Jamaica or Jordan or Kazakhstan or Kenya or Kiribati or "Korea, Democratic People's Republic" or Kosovo or "Kyrgyz Republic" or "Lao PDR" or Lebanon or Lesotho or Liberia or Libya or Madagascar or Malawi or Malaysia or Maldives or Mali or "Marshall Islands" or Mauritania or Mauritius or Mexico or "Micronesia, Federated States" or Moldova or Mongolia or Montenegro or Morocco or Mozambique or Myanmar or Namibia or Nepal or Nicaragua or Niger or Nigeria or "North Macedonia" or Pakistan or Palau or "Papua New Guinea" or Paraguay or Peru or Philippines or "Russian Federation" or Rwanda or Samoa or "Sao Tome and Principe" or Senegal or Serbia or "Sierra Leone" or "Solomon Islands" or Somalia or "South Africa" or "South Sudan" or "Sri Lanka" or "St. Lucia" or "St. Vincent and the Grenadines" or Sudan or Suriname or "Syrian Arab Republic" or Tajikistan or Tanzania or Thailand or Timor-Leste or Togo or Tonga or Tunisia or Turkey or Turkmenistan or Tuvalu or Uganda or Ukraine or Uzbekistan or Vanuatu or "Viet Nam" or "West Bank and Gaza" or "Yemen, Republic" or Zambia or Zimbabwe) |
| #12 | #10 or #11 |
| #13 | TI (Qualitative Research/ or Focus Groups/ or Interviews as Topic/ or Interview/ or Grounded Theory/ or Narration/ or Personal Narrative/) or (“Qualitative research” or “qualitative studies” or interview* or "focus group*" or “key informant” or hermeneutic* or heuristic* or "grounded theory" or narrative* or “thematic analysis” or "content analysis” or experienc* or ethnolog* or ethnomethodolog*) not TI (cross-sectional or “cohort study” or “case control” or “longitudinal study” or “retrospective study” or “prospective study” or regression*) or AB (Qualitative Research/ or Focus Groups/ or Interviews as Topic/ or Interview/ or Grounded Theory/ or Narration/ or Personal Narrative/) or (“Qualitative research” or “qualitative studies” or interview* or "focus group*" or “key informant” or hermeneutic* or heuristic* or "grounded theory" or narrative* or “thematic analysis” or "content analysis” or experienc* or ethnolog* or ethnomethodolog*) not AB (cross-sectional or “cohort study” or “case control” or “longitudinal study” or “retrospective study” or “prospective study” or regression*) |
| #14 | #1 and #9 and #12 and #13 |

**MEDLINE**

| Concept | Search strings |
| --- | --- |
| #1 | exp Social Support/ or exp Family Support/ or (support adj1 (social or famil* or friend* or spouse* or partner* or sibling* or relation* informal or informational or tangible or peer or friend* or organization* or functional or instrumental or material)).ti,ab. |
| #2 | Diabetes Mellitus/ or (“diabetes mellitus” or diabetes or “type 1 diabetes” or “type 2 diabetes”).ti,ab. |
| #3 | HIV/ or HIV Infections/ or Acquired Immunodeficiency Syndrome/ or (HIV or “Human Immunodeficiency Virus” or AIDS or “Acquired immunodeficiency syndrome”).ti,ab. |
| #4 | COVID-19/ or (COVID-19 or coronavirus or “coronavirus disease”).ti,ab. |
| #5 | Neoplasms/ or (neoplasm* or cancer* or tumor* or malignan*).ti,ab. |
| #6 | Stroke/ or (stroke or “ischemic stroke” or “cerebrovascular accident”).ti,ab. or (hemorrhage adj1 (cerebral or intracerebral or brain)).ti,ab. |
| #7 | Cardiovascular Diseases/ or (“cardiovascular disease” or “cardiac arrest” or “heart failure” or “myocardial infarction” or “myocarditis” or angina or arrythmia*).ti,ab. |
| #8 | Dementia/ or Alzheimer Disease/ or (dementia or “Alzheimer disease”).ti,ab. |
| #9 | #2 or #3 or #4 or #5 or #6 or #7 or #8 |
| #10 | ((Low$ or poor$) adj1 (income or resource) adj1 (setting or countr*)).ti,ab. or (resource$poor or resource$limited or resource$restricted or LIC or LMIC).ti,ab. |
| #11 | (Afghanistan or Angola or Bangladesh or Benin or Bhutan or Bolivia or Bulgaria or "Burkina Faso" or Burundi or "Cabo Verde" or Cambodia or Cameroon or "Central African Republic" or Chad or Comoros or "Congo" or "Democratic Republic of Congo" or "Cote d'Ivoire" or Djibouti or Egypt or Eritrea or Eswatini or Ethiopia or Gambia or Ghana or Guinea or Guinea-Bissau or Haiti or Honduras or India or Jordan or Kenya or Kiribati or "Korea, Democratic People's Republic" or "Kyrgyz Republic" or "Lao PDR" or Lebanon or Lesotho or Liberia or Madagascar or Malawi or Mali or Mauritania or Mexico or "Micronesia, Federated States" or Moldova or Mongolia or Montenegro or Morocco or Mozambique or Myanmar or Namibia or Nepal or Nicaragua or Niger or Nigeria or "North Macedonia" or Pakistan or Palau or "Papua New Guinea" or Paraguay or Peru or Philippines or "Russian Federation" or Rwanda or Samoa or "Sao Tome and Principe" or Senegal or Serbia or "Sierra Leone" or "Solomon Islands" or Somalia or "South Africa" or "South Sudan" or "Sri Lanka" or "St. Lucia" or "St. Vincent and the Grenadines" or Sudan or Suriname or "Syrian Arab Republic" or Tajikistan or Tanzania or Thailand or Timor-Leste or Togo or Tonga or Tunisia or Turkey or Turkmenistan or Tuvalu or Uganda or Ukraine or Uzbekistan or Vanuatu or "Viet Nam" or "West Bank and Gaza" or "Yemen, Republic" or Zambia or Zimbabwe).ti,ab. |
| #12 | #10 or #11 |
| #13 | (Qualitative Research/ or Focus Groups/ or Interviews as Topic/ or Interview/ or Grounded Theory/ or Narration/ or Personal Narrative/) or (“Qualitative research” or “qualitative studies” or interview* or "focus group*" or “key informant” or hermeneutic* or heuristic* or "grounded theory" or narrative* or “thematic analysis” or "content analysis” or experienc* or ethnolog* or ethnomethodolog*).ti,ab. not (cross-sectional or “cohort study” or “case control” or “longitudinal study” or “retrospective study” or “prospective study” or regression*).ti,ab. |
|  | #1 and #9 and #12 and #13 |

**PsycINFO**

| Concept | Search strings |
| --- | --- |
| #1 | exp Social Support/ or (support adj1 (social or famil* or friend* or spouse* or partner* or sibling* or relation* informal or informational or tangible or peer or friend* or organization* or functional or instrumental or material)).ti,ab. |
| #2 | Diabetes Mellitus/ or (“diabetes mellitus” or diabetes or “type 1 diabetes” or “type 2 diabetes”).ti,ab. |
| #3 | HIV/ or HIV Infections/ or Acquired Immunodeficiency Syndrome/ or (HIV or “Human Immunodeficiency Virus” or AIDS or “Acquired immunodeficiency syndrome”).ti,ab. |
| #4 | COVID-19/ or (COVID-19 or coronavirus or “coronavirus disease”).ti,ab. |
| #5 | Neoplasms/ or (neoplasm* or cancer* or tumor* or malignan*).ti,ab. |
| #6 | Stroke/ or (stroke or “ischemic stroke” or “cerebrovascular accident”).ti,ab. or (hemorrhage adj1 (cerebral or intracerebral or brain)).ti,ab. |
| #7 | Cardiovascular Diseases/ or (“cardiovascular disease” or “cardiac arrest” or “heart failure” or “myocardial infarction” or “myocarditis” or angina or arrythmia*).ti,ab. |
| #8 | Dementia/ or Alzheimer Disease/ or (dementia or Alzheimer disease).ti,ab. |
| #9 | #2 or #3 or #4 or #5 or #6 or #7 or #8 |
| #10 | ((Low$ or poor$) adj1 (income or resource) adj1 (setting or countr*)).ti,ab. or (resource$poor or resource$limited or resource$restricted or LIC or LMIC).ti,ab. |
| #11 | (Afghanistan or Albania or Algeria or Angola or Argentina or Armenia or Azerbaijan or Bangladesh or Belarus or Belize or Benin or Bhutan or Bolivia or "Bosnia and Herzegovina" or Botswana or Brazil or Bulgaria or "Burkina Faso" or Burundi or "Cabo Verde" or Cambodia or Cameroon or "Central African Republic" or Chad or China or Colombia or Comoros or "Congo" or "Democratic Republic of Congo" or "Costa Rica" or "Cote d'Ivoire" or Cuba or Djibouti or Dominica or "Dominican Republic" or Ecuador or Egypt or "El Salvador" or "Equatorial Guinea" or Eritrea or Eswatini or Ethiopia or Fiji or Gabon or Gambia or Georgia or Ghana or Grenada or Guatemala or Guinea or Guinea-Bissau or Haiti or Honduras or India or Indonesia or Iran or Iraq or Jamaica or Jordan or Kazakhstan or Kenya or Kiribati or "Korea, Democratic People's Republic" or Kosovo or "Kyrgyz Republic" or "Lao PDR" or Lebanon or Lesotho or Liberia or Libya or Madagascar or Malawi or Malaysia or Maldives or Mali or "Marshall Islands" or Mauritania or Mauritius or Mexico or "Micronesia, Federated States" or Moldova or Mongolia or Montenegro or Morocco or Mozambique or Myanmar or Namibia or Nepal or Nicaragua or Niger or Nigeria or "North Macedonia" or Pakistan or Palau or "Papua New Guinea" or Paraguay or Peru or Philippines or "Russian Federation" or Rwanda or Samoa or "Sao Tome and Principe" or Senegal or Serbia or "Sierra Leone" or "Solomon Islands" or Somalia or "South Africa" or "South Sudan" or "Sri Lanka" or "St. Lucia" or "St. Vincent and the Grenadines" or Sudan or Suriname or "Syrian Arab Republic" or Tajikistan or Tanzania or Thailand or Timor-Leste or Togo or Tonga or Tunisia or Turkey or Turkmenistan or Tuvalu or Uganda or Ukraine or Uzbekistan or Vanuatu or "Viet Nam" or "West Bank and Gaza" or "Yemen, Republic" or Zambia or Zimbabwe).ti,ab. |
| #12 | #10 or #11 |
| #13 | (Qualitative Research/ or Focus Groups/ or Interviews as Topic/ or Interview/ or Grounded Theory/ or Narration/ or Personal Narrative/) or (“Qualitative research” or “qualitative studies” or interview* or "focus group*" or “key informant” or hermeneutic* or heuristic* or "grounded theory" or narrative* or “thematic analysis” or "content analysis” or experienc* or ethnolog* or ethnomethodolog*).ti,ab. not (cross-sectional or “cohort study” or “case control” or “longitudinal study” or “retrospective study” or “prospective study” or regression*).ti,ab. |
| #14 | #1 and #9 and #12 and #13 |

**SCOPUS**

| Concept | Search strings |
| --- | --- |
| #1 | TITLE-ABS-KEY (support W/1 (social or famil* or friend* or spouse* or partner* or sibling* or relation* or informal or informational or tangible or peer or friend* or organization* or functional or instrumental or material)) |
| #2 | TITLE-ABS-KEY ("diabetes mellitus" or diabetes or "type 1 diabetes" or "type 2 diabetes") |
| #3 | TITLE-ABS-KEY (HIV or "Human Immunodeficiency Virus" or AIDS or "Acquired immunodeficiency syndrome") |
| #4 | TITLE-ABS-KEY (COVID-19 or coronavirus or "coronavirus disease") |
| #5 | TITLE-ABS-KEY (neoplasm* or cancer* or tumor* or malignan*) |
| #6 | TITLE-ABS-KEY (stroke or "ischemic stroke" or "cerebrovascular accident") or (hemorrhage W/1 (cerebral or intracerebral or brain)) |
| #7 | TITLE-ABS-KEY ("cardiovascular disease" or "cardiac arrest" or "heart failure" or "myocardial infarction" or "myocarditis" or angina or arrythmia*) |
| #8 | TITLE-ABS-KEY (dementia or "Alzheimer disease") |
| #9 | #2 or #3 or #4 or #5 or #6 or #7 or #8 |
| #10 | TITLE-ABS-KEY ("Low* income" or "poor* income" or resource*poor or resource*limited or resource*restricted or LIC or LMIC) |
| #11 | TITLE-ABS-KEY (Afghanistan or Albania or Algeria or Angola or Argentina or Armenia or Azerbaijan or Bangladesh or Belarus or Belize or Benin or Bhutan or Bolivia or "Bosnia and Herzegovina" or Botswana or Brazil or Bulgaria or "Burkina Faso" or Burundi or "Cabo Verde" or Cambodia or Cameroon or "Central African Republic" or Chad or China or Colombia or Comoros or "Congo" or "Democratic Republic of Congo" or "Costa Rica" or "Cote d'Ivoire" or Cuba or Djibouti or Dominica or "Dominican Republic" or Ecuador or Egypt or "El Salvador" or "Equatorial Guinea" or Eritrea or Eswatini or Ethiopia or Fiji or Gabon or Gambia or Georgia or Ghana or Grenada or Guatemala or Guinea or Guinea-Bissau or Haiti or Honduras or India or Indonesia or Iran or Iraq or Jamaica or Jordan or Kazakhstan or Kenya or Kiribati or "Korea, Democratic People's Republic" or Kosovo or "Kyrgyz Republic" or "Lao PDR" or Lebanon or Lesotho or Liberia or Libya or Madagascar or Malawi or Malaysia or Maldives or Mali or "Marshall Islands" or Mauritania or Mauritius or Mexico or "Micronesia, Federated States" or Moldova or Mongolia or Montenegro or Morocco or Mozambique or Myanmar or Namibia or Nepal or Nicaragua or Niger or Nigeria or "North Macedonia" or Pakistan or Palau or "Papua New Guinea" or Paraguay or Peru or Philippines or "Russian Federation" or Rwanda or Samoa or "Sao Tome and Principe" or Senegal or Serbia or "Sierra Leone" or "Solomon Islands" or Somalia or "South Africa" or "South Sudan" or "Sri Lanka" or "St. Lucia" or "St. Vincent and the Grenadines" or Sudan or Suriname or "Syrian Arab Republic" or Tajikistan or Tanzania or Thailand or Timor-Leste or Togo or Tonga or Tunisia or Turkey or Turkmenistan or Tuvalu or Uganda or Ukraine or Uzbekistan or Vanuatu or "Viet Nam" or "West Bank and Gaza" or "Yemen, Republic" or Zambia or Zimbabwe) |
| #12 | #10 or #11 |
| #13 | TITLE-ABS-KEY ("Qualitative research" or "qualitative stud*" or interview* or "focus group*" or "key informant" or hermeneutic* or heuristic* or "grounded theory" or narrative* or "thematic analysis" or "content analysis" or experienc* or ethnolog* or ethnomethodolog*) |
| #14 | TITLE-ABS-KEY (cross-sectional or "cohort study" or "case control" or "longitudinal study" or "retrospective study" or "prospective study" or regression*) |
| #15 | #13 AND NOT #14 |
| #16 | #1 AND #9 AND #12 AND 15# |
