# Supplementary material for: Patients’ and caregivers’ experiences of familial and social support in resource-poor settings: A systematically constructed review and meta-synthesis
Source: Palliat Care Soc Pract. 2025 Jun 27;19:26323524251349840. doi: 10.1177/26323524251349840 (PMC12205196; doi:10.1177/26323524251349840)
Supplement: sj-docx-2-pcr-10.1177_26323524251349840 – Supplemental material for Patients’ and caregivers’ experiences of familial and social support in resource-poor settings: A systematically constructed review and meta-synthesis [file sj-docx-2-pcr-10.1177_26323524251349840.docx]

**Qualitative quotes on familial and social support**

| **Author (Year)** | **Condition** | **Verbatim quotes** |
| --- | --- | --- |
| Adam & Koranteng  (2020) | Cancer (breast) | *“In terms of financial support I used to get support from my coworkers. This person will send and this person will send but now I feel shy to tell them again.”*  *“Apart from myself, in terms of financial support, I get support from my husband, my sister and my mother. They have been of great support.”*  *“No support. I am the who pays, my daughter goes with the money to buy the things like injections.”*  *“I am the only one who pays so when it happened like that, I wanted to build a house so I sold everything and use the money to help myself.”*  *“We are told to eat balanced diet every day, in the beginning I used to do as they told but now I don’t get it like so I’m forced to eat whatever food I get and my daughter has been helping with the house chores.”*  *“I do all my home activities by myself.”*  *“No I don’t get help from anywhere, as for food no one helps me.”*  *“Okay when I came for the surgery and left, church members and friends came to visit. My family keeps me company.”*  *“Only a few people know I have cancer, but for my sister and my children who know I have cancer they show me love and concern.”* |
| Adejoh et al. (2021) | Cancer | *“I take care of her. . . just like I went to the store to help her there, then after I come back from the store I still help her at home. Whenever she is feeling pain I will ask her what is wrong with you she will tell me she is feeling pain, then I will ask her to take drugs so that the pain will come down. Sometimes she will tell me that no, I will still plead with her that she should take the drugs, she will take the drugs, sometimes she will be vomiting, I will still run to her, give her water I will be telling her sorry, sorry so that she will feel better and be happy.”* |
| Adejoh et al. (2024) | Cancer (breast) | *“My husband has been nice. I have never seen a man like him. He takes me to the hospital each time I need to go. Even when I was doing radiotherapy, you won’t believe that this man leaves the house as early as 03:00 am to EEE hospital [not real name of the hospital] just to put down my name in the register so that I’ll be one of the first persons to be attended to by the doctors. There’s no medicine or food that is recommended by the doctor that he doesn’t buy for me. He even buys and blends fruits for me to take. In fact, he’s one in a million.”*  *“My relatives have been wonderful. Ah! I never knew they loved me like this. Especially one of my brothers. In fact, I said for the sake of this my brother, God should allow me live. He wanted taking me out of the country for my treatment, but my instinct told me to do it here in Lagos. He’s been supportive. Any money that I mentioned, he’ll tell my husband to split it into two and he’ll contribute half of it for my hospital bills. He’s been nice and caring. They wake me up every morning with prayers. I never knew they loved me this much until I became sick. I thought that with this sickness, things will be difficult but to the Glory of God, things are even becoming better. God is giving my husband business links. God has been so faithful to me.”*  *“I used to be a Sunday school teacher and a member of women missionary group. They support me financially, visitations, prayers, and advice. There’s a woman in my church who has been a breast cancer survivor for 25 years that encourages me to be strong and I am really coping.”* |
| Alqaissi & Dickerson (2010) | Cancer  (breast) | *‘‘At time of chemotherapy, my sister and my sister-in-law used to do everything for me. They cooked for me, they gave me bath, and they took care of my daughter.’’*  *‘‘When I got the disease, he (husband) told me, you deserve it because you have extreme hate inside. God is punishing you. He beat me, he hit me with a chair, he suffocated me, and threw me out of the home.’’*  *‘‘I did my surgery in a private hospital. It cost me 2000 dinars. All people participated, my daughters, son, and friends.”*  *‘‘A friend of the family offered money to my husband after I got the disease, although we don’t need the money, but this shows you how much people care for you.’’*  *‘My cousin is a breast cancer survivor.... She tells me what’s going to happen step by step.... She told me you have stage II, and yours is early so you will be fine. She is giving me a huge emotional support.’’*  *‘‘My nephews live in [country name]. They are rich. They called me and offered treating me outside the country.’’* |
| Bahrami et al. (2014) | Heart failure | “Although we hospitalized him regularly, but my other family members little know about our financial problems ... I had to take a loan for buying his heart machine ... I feel that I cannot follow the treatment process in the future”.  “My relatives and neighbors seldom ask about our situation. they ask and help me earlier ... Now everybody has several problems in his or her life ... my main problem is just financial now.”  “We are tired of spending so much money to receive the treatment ... he has been hospitalized continuously ... The government should pay more attention to us by giving more financial support and discount on the hospitals costs.” |
| Banchani et al. (2020) | Non-communicable disease including hypertension, diabetes, and stroke | *“Because it is my husband who gives me money to go to the hospital, even if I am lying down and I can’t get up, he is the one that will cook for me. He gives me my drugs to take. And my children also cook for me. They are young, so they can’t help with money, so it all boils down to my husband. He is the one who does everything for me. And excuse my language, but if I want to defecate, he holds my hands and takes me to free myself and he also bathes me because I feel pains in my legs. So, as for my husband, he is the one who does everything for me. Even when I am coming to the hospital, he is the one who brings me in his car.”*  *“I live with one of my grandchildren. So, those who don’t live with me, when they come, they encourage me with words.... When they come, I tell them that I am worried about this illness and they will say “it will be over, Mummy. It will stop, it will stop okay.” This comforts me.”*  *“They ask me “Mom, have you taken your medication. Mom, how are you?” Some will come and greet me “Maame, how are you,” or “Grandma, how are you?” I will tell them by God’s grace I am fine.”*  *“Yes, I get a lot of appraisal support. People tell me that many have had it [stroke] and the Lord has taken them through. I am not the first and I should not be the last person. So, I shouldn’t think that it is only me or it is a curse from somebody.”*  *“Right now, I have the support of my wife and the children. They are the sole persons I’m closer to. They took care of me in the greater part of my ailment.”*  *“My brothers and sisters, they give me that support a lot. Sometimes they bring me money and then buy the prescribed drugs and the food that I take. You know, we diabetes patients, we take a lot of vegetables. So, they buy all for me. Sometimes fruits.”*  *“Yes. That’s my wife. I trust her. From the beginning, she felt that, whatever situation that I am in, she is with me, so I should not worry. Secondly, she has been telling me that, from her dreams, she knows that I will become better and actually she saw to the progression of my recovery, and it looks like her dreams are materializing.”*  *“Because when I was attacked by this sickness, I was bed ridden. I am currently not working. So if I’m visiting the hospital, it is my children who give me money...the children too are not in any gainful employment, so when they prescribe the medicine for me, sometimes, I don’t get money to buy them.”* |
| Biney et al. (2024) | Cancer | *“My cousin*,*who is a doctor*,*helps me a lot. She takes her on hospital rounds sometimes. My cousin*,*my uncle*,*and my husband have helped a lot*,*especially my cousin. When we come to the hospital and there is a drug to buy*,*she just picks it up and buys the drug. Sometimes I don’t ask for money*,*but they just give it like that. The one that surprises me is even my sick mother. … I don’t want to talk much*”.  “*My children and husband support me by attending to my father when I may be doing something. Sometimes I instruct my children to do other things*,*and they are always available. I have observed for some time now*,*they (*children) *stay at home and even ask Mummy*,*“Can I help you with something?“*  *“Like I said earlier*,*our doctor at the hospital is also our pastor at church*,*he has been supporting us… he helps in prayers*,*he has informed the church about it and requested they pray for me and my husband all the time. The financial support is coming from the pastor and the church as well”.*  “*Every Friday evening*,*our Imam visits us at home to offer prayers for me and my sister. Sometimes he recites the Quran*,*and other times when he is unable to come*,*he sends a representative to come to us on his behalf. His representative will always encourage me that Allah is in this*,*so I shouldn’t worry (*she burst into tears and smiled a little after*) At times*,*some of the believers come to see us when we are home*,*especially on Fridays*,*to encourage us.”*  *“… the chief Imam in our community organizes regular prayer sessions for my father in our house whenever he is called upon.”* |
| Binka et al. (2019) | Cancer (cervical) | *“I gave her all the necessary things that she needed. Sometimes, I borrowed money; sometimes too, I had to sell my things. Luckily enough, her employers also came in and helped her a lot.”*  *“I assisted her financially and when it came to cooking, I gave money to the kids to take care of that. Also, I had to take a casual leave to assist her in managing her shop and accompany her to the hospital for treatment”.*  *“Because of the disease, my husband left me and went to marry another woman. So we are no more together. Only my child is helping me”.*  *“I have no support; I do everything on my own even though I was advised by a nurse not to lift heavy things after I was discharged from the hospital.”*  *“Nobody provided any support for me. I remember there was a time I even needed 300 Ghana Cedis for my treatment, and nobody helped me. Even the church I was then attending disappointed me.”* |
| Brown et al. (2022) | HIV | *“I did not want to visit him at all, but my wife pushed me to do that. Each month the money I had to spend two million vnd (~$90) to visit my son and it was almost the same as the amount of my monthly salary.”*  *“[My family] visited me once every two or three months. Each time they came they brought canned foods (meat, fish) and peanuts because there was nothing to eat with white rice there.”* |
| Duodu et al. (2024) | Dementia | *“He is our father so when his relapse starts, any of us comes to help him. Even if we are asleep and another is awake, he that is awake calls for us that Baba’s condition has started. We all come together and help him. When I am away, my siblings are the ones who take care of him. No one speaks to him impolitely or harshly because we know it is a sickness and also that was not how he was at first.”*  *“The family tries to support; for those that are not here would call and ask of her. She could mention her children’s names and we would call them for her to say good morning. She is able to respond to greetings and salutations. Her grandchildren sometimes come around to talk to her even though she would not understand. Her loved ones also pass by sometimes.”*  *“I do not have any financial challenges when it comes to her because my brothers send money every week. Those in Accra [capital city of Ghana] send theirs every month, so when it comes to struggling financially, that is not the case.”* |
| Hamid et al. (2021) | Cancer  (breast) | *“My mother is nearly 65 but she stayed with me when I was admitted to the hospital for surgery. After being discharged from there, she took care of all my needs at home. As I could not stand on my own after surgery, she would take me to the bathroom, help me in taking shower, and make me eat food with her hands. I have seen her crying for me in prayers. If I ever was in pain at night, she would stay awake and take care of me. That was a real comfort.”*  *“My husband was really nice throughout just about everything. After first surgery, I was unable to do any work at home or take care of my children. My husband assumed the role of a father as well as a mother. He would do all the household work after reaching home by evening. For those two months, he even brought food for me to my bed. All I can say about him is that he is the biggest support system that I could have ever expected.”*  *“When I learnt about my illness, I was devastated and was about to burst into tears. My father, who accompanied me to the hospital, hugged me and kissed my forehead. He is a laborer and his earnings hardly suffice our daily needs. We had a small piece of land which he sold for my treatment. Whenever I ask him about it, his answer is always the same, ‘you are more important to us than anything else’. That is a really great support. His words always encourage me and give me a different kind of relief.”*  *“After the divorce, I lived happily with my parents. They would take care of me and my children. They even managed the costs of my treatment and never left me alone. After their death, I had no choice but to stay with my brother. Initially he was very good to us and took the responsibility of my treatment. But a few months later things started changing, and he asked me to leave the house. I was sick and in need of care and support. But he was never concerned about our wellbeing or my illness. This hurts me a lot.”*  *From the day of my cancer diagnosis, I witnessed a change in my husband’s behaviour. He never listened to me whenever I tried to talk to him about my illness. He was getting very rude with each passing day. I always wanted him to be there with me for all my doctor’s appointments, but he never did. My expectations were really hurt.*  *“My husband was always by my side. When I had to go for first chemotherapy, I was very nervous and talked to him about it. He held my hand and told me, ‘You will be fine’. He was very supportive and caring all the time. He makes me feel complete. His love and support helped me to accept my condition and fight with the disease.”*  *“Because of chemotherapies, I am always tired and find it difficult to keep up the pace I once did with housework, and mothering. Now my eldest daughter takes care of the housework and at the same time she takes care of me and her siblings. In order to run the house and manage my costly treatment, my son left his studies and joined a private company. If I am alive, it is only because of my children’s love and support.”*  *“At times the problem of transport in our area turns out to be a very challenging issue, especially when I have to go to the hospital. Fortunately, my father’s cousin owns a car. When I have to visit the doctor, he offers us the ride. If we ever ran out of money, he would help us by paying the hospital bills. I really feel good when I see my relatives taking care of me and my children in such difficult times.”*  *“It was the support of my friends that got me through my illness and surgeries. When they learned about my illness they came to visit me one by one. They keep coming to my home even today and bring gifts and ‘get well soon’ cards along. I feel amazed when I talk to them. They are a wonderful source of encouragement and support.”*  *“I shared a very good relationship with my friends. But when my illness was detected, a few of them stopped interacting with me. When I called them myself, I was often told: ‘I was going to call you’, ‘I was so busy at my home that I was not able to come and meet you’, and so forth. However, their statements and explanations did not help me in anyway. Rather, I felt neglected. Whenever I think about it, I get hurt.”* |
| Hendricks-Lalla & Pretorius (2020) | Dementia (Alzheimer’s disease) | *“It is very taxing to care for my wife without emotional support from my daughters. It is actually very sad for me because I thought that my children would support me but they disappoint me. They don’t come forward...My wife’s sister and mother also have Alzheimer’s disease and their families do not support them. I wanted it to be different with my wife. I wanted our children to be there for her.”*  *“The most challenging part is the support...I don’t get support from any family member. Her siblings totally discarded her......”*  *“The two daughters till today is withdrawn and the youngest one is only involved. We do not hear from the two in Cape Town. They stay away. They don’t phone to ask how she is.*  *“I never knew how to cook, besides frying eggs. Now I can make all kinds of bredies (stews). I feed my wife, dress her, take her on walks, supervise her and cut her nails...basically I do everything.”*  *“I thanked her (my sister) for taking Mom for a couple of days. I had vacation at that time without realising it. It was a relief for me.”*  *“The love that I have for my wife. Knowing the person that she was. I also have a sense of duty to care for her being her husband and protector.”*  *“He (God) turned the wheel the other way; it was pay-back time. This is now my time.”*  *“My ‘deen’ (religion) carries me through difficult times and makes it easier to fulfil my role. My belief in God carries me through. I have accepted it and try to make the most of the time I have with my beloved.”* |
| Hesamzade et al. (2017) | Stroke | *“I take him to physiotherapy clinic and teach him how to do physiotherapy exercises at home under the physiotherapist’s guide”*  *“I prepare food for my mother and gave the food by spoon to her.”*  *“When I am not with him, I give him a ring and talk to him on the phone to prevent him from feeling of being lonely.”*  *“I take her to the bathroom and helped her to take shower.”*  *“We (family members) built a bathroom and a toilet next to her room and she can easily go to the lavatory or take a shower.”*  *“When my father got sick (stroke), we have not any financial problem because my brother manages his farm and garden and gave the*  *benefits to my father.”* |
| Hobenu & Naab  (2023) | Cancer (cervical) | *“My husband tells me to be courageous. He also said that whatever God does is at its apportioned time, this disease will go and we will be happy again.”*  *“He [husband]...cares for me. He accompanies me to the radiotherapy centre...He massages my legs every evening.”*  *“My husband has done very well...he is always praying for me to get well.”*  *“My husband has been my only source of support financially. He...provides all the money I need for my treatment and other needs.”*  *“My siblings...contributed money for me. Had it not been for my family members and my children who supported me financially, I would have died. Yes, they helped a lot.”*  *“My children are there to support me domestically. They sweep, fetch water, cook and clean the house...I don’t know what I would have done without their support.”*  *“The time the sickness was serious, I stayed at home for months...Even though I could not go to work...my employer paid my full salary and my hospital bills...Some of my co-workers call to check up on me...Whenever my manager cannot call me directly, he finds out from my close friend...how I am doing.”*  *“My husband has never given me money...for my treatment. How can I be calling this man my husband?...I wake up in the same room with him, and after dressing up to go to the hospital, he will only bid me goodbye...Sometimes, by the time I finished bathing, he had left the house without my knowledge because he wanted to avoid the situation where I would ask him for money for the hospital. It is not easy for me.”*  *“My family is aware I am sick, but none has ever assisted me financially...none of them cares about me. They said...it is my husband’s responsibility to look after me when I am sick.”* |
| Jabeen et al. (2024) | Cancer (breast) | *“I bring her here, as it is my responsibility to take care of her as an elder son. Though it is a truth that at the moment I don’t have any other option, but I believe her prayers and blessings from the people around are big reward that will pay back to me in some form.”*  *“Ever since we told her husband about her breast cancer, he has not returned from Saudi Arabia. He has not even tried to contact his wife after that. He couldn’t bear the financial burden of treatment.”*  *“She is my sister. I can’t throw her out of home like her husband. I have a strong blood relation with her, so it is a compulsion on me to take care of herself. Although my physical and mental health has been adversely affected in this situation.”* |
| Knight &Schatz (2022) | HIV | *“I am happy here because the people living in my community are warm people, we help each other, even when I started becoming ill, they used to be very helpful. They brought me food and things that I needed.”*  *“I am being cared for in all ways and my daughter reminds me even if I am in bed and would say “father, father, here are your pills. . .father, father have you eaten already” and then I would ask her to bring my pills to drink.”*  *“When I am very ill my children come and fetch [medication package] on my behalf.”*  *“Yes, even when I have to go to Tygerberg one of [my sons] because two of them have cars, will transport me and will phone me while there to find out how far [I am and if] I have been attended to and then when [I’m] finished I’m fetched again.”*  *“[My son who lives away and works] would say “no don’t buy groceries just pay the other things, I’ll do the groceries and other things and I do buy clothes. It’s just at certain times because his mother is alive, and the person I live with is not his mother, so he comes at intervals and then goes to live with his mother again. .. I tell him to buy items and not give me money.”*  *“[they support me] very much and even when I was sick, they were here when I was sick last year, they all came out here and checked on me at the hospital; I was admitted for a week and the second week I was discharged.”*  *“My sister told me this is a health problem that is found in people and that I should not be thinking that it is me alone, I must withstand it because I will also get better just like other people.”* |
| Kusi et al. (2020) | Cancer (breast) | “She is my mother, my family… it is actually my socio-cultural responsibility to take care of her… That is the reason I am the one taking care of her.”  “As our culture demands, I have to take care of her. It is just my duty as her (patient) family member.”  *“Hmmm..! As the eldest daughter, it is my cultural duty to take care of her. I have no other option than to take care of her”*  *“I am a woman and her mother and it is my cultural duty to take care of her.”*  *“Oh! She is my wife… if I don’t take care for her, who will? It is just my social obligation as a husband to take care of her.”*  *“When she was diagnosed of the disease (breast cancer) she had no one to take care of her, hence, as a brother, it is culturally expected that I take care of her. There are no women around, hence I have to do it.”*  *“It is unusual for someone who is not your family member to take care of you when you are sick, but she (patient) did it passionately when I was admitted at the hospital. So now that she is sick, it is my turn to repay her for what she did for me.”*  *“She used to support me by lending me money when she was working. So now that she is sick, I have to also support her till she recovers from this illness. I am just paying her back for all the support she has been giving me.”*  *“I fetch and boil water for her every day. I also groom her every day because she becomes very weak whenever she goes for therapy.”*  *“She cannot wash her dirty cloths because her hand is always swollen and heavy (lymphedema). So, I am the one who does all her laundry.”*  *“I pray for her and share healing messages in the Bible with her. This has really helped her to have some inner peace now.”*  *“Now she (patient) does not cry anymore because I always encourage her that God is on the throne and that He will heal her. I pray and share God’s words with her. These have really increased her faith in God.”*  *“Sometimes, I arrange pastoral visits for her. The Osofo (Reverend) comes in to pray with her.”*  *“She was always thinking about the disease, but I encourage her with cheerful and hopeful messages to relieve her emotional distress.”*  *“I always make sure that I communicate with her and encourage her to forget about the breast cancer so that she can be happy all the time.”*  *“Although I worry a lot about her condition every day, I try to be cheerful when I am with her. I get time to listen to her and console her when she is lonely.”*  *“The National Health Insurance Scheme (NHIS) does not cover all the cost of the treatment. So, the little I get, I spend it all on my mother’s drugs and living expenses.”*  *“I provide money for everything including transportation to the hospital. Where we live, if we take a taxi to the hospital, they charge GHC 120 (≈23 USD) for our round trip.”*  *“Aside money for her drugs, I always buy food supplements (Forever Living) to meet her nutritional needs. Although expensive, I manage to buy it for her.”* |
| Lelaka et al. (2022) | HIV | *“My mom understands and accepted me and my relationship, she helps me a lot with my younger child, and she is my pillar of strength.”*  *“My sister and my mother love and support me so much. They check on me constantly, regarding my pre-exposure prophylaxis medication, health and how I am coping. They ask a lot of questions about me and my health and not about my partner. Even me too, I tell them how I feel, and they reach out to me when I need their support or help. They also love my family, that is my partner, and my children. I feel welcome when I visit my family, and I am not scared. I feel at home.”*  *“In general terms, my parents and sister do provide me with support, and we are all in good terms. They are normally there, and they have been there for me. They have ensured my health is taken care of.”*  *“My friend also knows about my HIV status, and she supports me so much and encourages me to stay positive.”*  *“Mom sometimes gives me money for taxi fare when accompanying my partner to the clinic for consultation/treatment.”*  *“She is responsible for a lot of things in the family, like taking care of the children, loving and supporting us all the time, fix things that are not going well, doing the washing and when Igo to work, I am clean.”*  *“My mom understands the situation and my relationship, she helps me a lot with my younger child, and she is my pillar of strength. She takes care of my child, and I don’t pay her. The other thing is that mom helps me with house chores particularly when I am not feeling well. I am happy with her continued support.”*  *“With the family, everyone supports me, and they remind me of taking medication, even now my mom knows I’m coming to Limpopo and she just told me that I must not forget my medication.”*  *“She reminds me to take my medication most of the time; I am so happy for the support that I get from her.”*  *“I (partner) accepted and supported her. I am happy with that. After testing HIV positive, I provided her with a lot of support, and she appreciated the support.”*  *“Even now (partner), he is sometimes supervising to ensure that I take my medication well and also to ensure I attend my visits.”*  *“He (partner) has been very helpful to the kids and me. He has been very hands-on with the house chores although he’s not a good cook he tries his best. When it comes to packing things in the house, he is very good as he ensures the house is neat. He is very helpful, and he assists me so much. He also cleans the house. He is good. I can rely on him in so many things, and he is not disappointing. He delivers.”*  *“My partner is so supportive. Sometimes it’s quite hectic at home when we have events, but he is there to support me. I do not feel any stress as he is doing a lot of things so because of him, I do not usually get stressed at all.”* |
| Mbozi et al.  (2023) | Cancer | *“I worry about what they will eat because at least when am home,.....a lot of bills at home, I have to buy water and pay for electricity,*  *just a lot.”*  *“Money is a problem for food, for example, when some have been discharged, they leave food for me sometimes money. Like today I was given a k50 buy food from a well-wisher.”*  *“The experience is okay, because when I look back, he also used to take care of me when he was working so now that his sick, they is nothing I can do but take care of him, am the only one that can do that because he’s not able to do that on his own.”* |
| Mbozi et al. (2023) | HIV/NCD | *“My wife helps me in a lot of ways. If I do not have money, she is the one who goes to loan money from people in our community who have it for me to get to the clinic; if there is something that I need if she can do something she will.”*  *“My daughter in-law, daughter, and sister-in-law, they all work together to ensure that I stay well. My daughter in-law is usually the one who does most of the care work for me. My sister wife helps here and there.”*  *“If I am not feeling well, I will call her [my sister] to come and help me. Maybe she will even accompany me to the clinic. She will help me in the house by cleaning, cooking, and washing my clothes.”*  *“When I am not feeling well, I just sit, and if my niece realises that she has not seen me for 2 or 3 days she comes to check on me. I explain what the matter is, and she assists me in the way she can.”* |
| Mlaba et al. (2021) | Cancer | *“[I] don’t have any support needs because the kids are around, and they are very supportive and helpful.”*  *“Oh no* *I had good friends that supported me and my neighbour, she would sleep over, to give me a break ... My family like I said, my son moved in with me to help me, gave up his place to be here to support me as he could and then if she got really sick ... her friends pick up the phone, they come flying down here take her to hospital.”*  *“[W]e were able to get all the help that we needed from the government hospital [Greys hospital], transporting her [patient] to Albert Luthuli hospital ... The hospital [Greys hospital] provided everything for her to go there, everything for her to get operated, she stays there and she is okay until she comes back, even when she came back from Albert [Inkosi Albert Luthuli Central Hospital] they didn’t take her back to Greys [hospital]. They brought her back home, so everything was taken care of by the people from Greys [hospital].”*  *“They [rest of the family] used to put in a lot of money but when it comes to helping physically with their hands they wouldn’t help, but money, financially she [patient] had everything that she needed and she always got it on time, even food if she wanted something today she would get it today on time but love, they [rest of the family] didn’t have it, they always had excuses about work ... No one cared how I felt and how hard it was for me, it was better if someone would come home from work and ask me how it was today, was it difficult or not you know.”* |
| Mohammadian et al. (2023) | Cancer | *“My younger brother contradicted us in my father’s treatment and hence, we gave up caregiving and delegate all caregiving-related responsibilities to him.”*  *“My family members are away from us. I would not be so alone in caregiving to my mother if they were near us.”*  *“The National Relief Foundation gives us $18 per month which is very little to solve any problem.”*  *“Each organization independently provides its services through its own pathways. Thus, one patient may simultaneously receive considerable help from several organizations, while another patient may receive limited help, if any. Close collaboration among these organizations could better organize their help and support services.”*  *Religion tells me “As you provide help, you will receive help”. Moreover, empathy and commitment towards each other are valuable principles in our family and all of us adhere to them. These things have caused us not to leave alone my wife in caregiving to her mother.”* |
| Mokhtari et al. (2022) | Cancer  (breast) | *“After my disease was diagnosed, we did more recreational activities because I was told that I should keep my spirit up to get better. My husband took me traveling and it made me feel better. Every-body was trying to keep me entertained, to make me happy, and to stop me from obsessing over my disease.”*  *“After my diagnosis, my husband supports me a lot and this made me have a better view of life. I decided with myself to prioritize my family after this, not my job; appreciate my health and that of my family and care about our fun and being together. Now I appreciate life more than before.”*  *“After the operation and mastectomy, the family supported me and i saw that life has its own beauties that i passed by very simply during several years of my life. When i looked at people’s faces, even the street had a different meaning to me. It was as if I had been born again. This led me to set good goals for myself and my children and I try to reach them.”* |
| Moyer et al. (2014) | HIV | *“Before they knew what the cause of the disease was, they were very concerned and would look after the person and try to find out what was the problem. But when it was discovered you were HIV positive, they knew you were dying, no treatment, they just abandoned you or took you to a rural home to die there.”*  *“The truth is there was a time when people were very sick; they had prolonged diarrhoea till they were finished. People were very scared of them, feared them; family members would abandon them and neighbours just came to look at them from afar, they would not touch them. It took a Good Samaritan to tend them; sometimes they used to die in the houses.”*  *“Those bedridden were locked up while family members went to work, or they just refused to offer them nursing care.... I went to some houses and found the bedridden patient was locked in from the outside...had not been washed, had soiled his beddings and vomited on the floor.... Houses used to stink.... We used to wash them and clean them. Some NGOs came and would pay us, but we also felt it was our responsibility to do that because most of us had recently been resurrected because of the care we received from other people infected with HIV. Our own family members feared touching us, so if no one came to your assistance you would stay in soiled linen.”*  *“There were those [sick people] who were alone in the houses.... We had some people from the Catholic Church who used to come to bathe them, clean their houses and wash their clothes, cook for and feed them, even cook for the children, and give them medicines.”* |
| Mphasha et al.  (2022) | Diabetes | *“My family knows that I’m diabetic and are supportive. They cook for me. They let me exercise by sweeping the floor since I’m in my 90s but supervise me to ensure that I don’t overwork myself.”*  *“I live with my wife. She cooks for me. When she is happy, I get happy with her. When she is emotional, I just avoid her so I can avoid stress. I have erectile dysfunction and the*  *wife understands. She is also too old; she no longer*  *desires sex that much like before.”*  *“My husband doesn’t fully support me, sometimes we always fight over salt-free dietary changes, and when I’m engaged and cannot manage to go to the clinic for collection of medication, he cannot collect it on my behalf. I instead request neighbors to do so, since our children are at school or work.”*  *My family knows I have diabetes and gives support, however my husband was being difficult and not supportive, when I first got diagnosed with hypertension and then diabetes I was pregnant, and I was trying to prepare family meals without salt, but my husband always insulted and tortured me as if I’m the one who chose to have the disease and I got stressed and admitted..”*  *“When I tell my wife that the food, she gives me to eat will kill me, the wife will instead reply by saying if you die, you die. This treatment started when I was no longer employed, though I built her a house”.* |
| Musyimi et al. (2024) | Dementia | *“I love caring and supporting my mother. Whenever she falls sick, I would rather lose my job but go and nurse her. For me, taking care of my ailing mother is a golden opportunity I have. Mother is ‘gold’ to me. I talk about her with a lot of love and compassion!”*  *“I felt a lot of sympathy inwardly. I used to work at secondary school [name withheld] and I decided to quit my job to come and take care of him. I got that inner feeling of sympathy that this is my father, and I needed to take good care of him. I quit my job completely to seriously see him get treated.”*  *“We might end up having Dementia when we age and so I handle Mother very well.”*  *“Africans fear that if our parents died because of what is believed to be negligence, they might curse us. In this case we give everything that we have to take care of them, not because we love them as much, but because we are afraid they might curse us and have negative impacts on our lives. Thus, it plays a lot of impact in our society.”* |
| Mwendwa et al.  (2021) | Dementia | *“In terms of food, we are ok; there is no problem with food here. We get support from the children; they are the ones who buy food for us.”*  *“The children have been very supportive with talking to me and encouraging me, and I thank God for them. I do not take this for granted because I know many children who do not support their parents even under normal circumstances.”*  *“...I have sisters, and none of them helps me. They do not even visit us (the person living with dementia says ‘they never come’). I usually ask them to send me at least a little money. They don’t seem to care. They always say they will come, but they never show up, never, and I am telling the truth.”*  *“......I have employed someone to take care of him. So he lives in XXX, and I live around here. But I basically handle a lot of stuff, his medication and his general welfare. I am the one who is mostly in charge.”* |
| Najjuka et al. (2023) | Cancers | *“I wake up, if he wants to eat, I get him breakfast, then I see what he wants to eat for lunch. Sometimes I prepare juice. I wash, I bathe him when he is weak, when he is unable to move, and we use a bucket.”*  *“Early in the morning, when Mzei [my father] has woken up, …I clean him in the mouth and then the body, and because he cannot move out, I dress him from here, so I have to remove the pumpers and change him into clean ones. Now when he is clean, I lay the bed …silence… after that, I then start to look for what to feed him, …. I feed him. Sometimes I massage him to make him feel relaxed. After I give him his medication then I wash his clothes.”*  *“One of the major activities I do are: to move Mzei around, if he is supposed to go to another ward, or at the OPD, I have to take him on a wheel chair and I move him around. When it is time for waiting for results, I have to make sure that I keep around to get the results. When we have been told to buy drugs, which are not inside here [UCI], it’s me who moves out to buy the drugs for him.”*  *“I had a financial package which I was given as my contract was ending… so right now, I have used all of it and I had some good harvest of maize as of last year’s second season, so that’s what am now selling. I also sold my cow to able to take care of my father.”*  *“… I have family members, church members and friends who are helping me. Church members have come home, like three or four times. They come as a group, and they sit with us, whatever they have, they give it to us. Also, our friends who are sending us money… those with money come at home and give us what they have.”*  *“It is tiresome but I am not taken up because I know why am doing all this. I get physically exhausted, but I do not get disgusted with the whole care giving experience. Sometimes the journey the pharmacy outside the hospital is long… that can be tiring.”*  *“If someone [relative] sends money, they think that they have assisted with care giving… fine, it helps, but it is not similar to you who is there all the time. They [relatives] were thinking that care giving is a walkover, so I also got annoyed as a normal person, and I said, fine, our father is there; you also help him. I left everything to them. I told them, from today, don’t even call me because I have left him for you. Get your own car, I have suspended my car because they had reached the level of thinking that the car is for daddy. So, I left him to them. Last Friday they had given up and said that they don’t have money.”*  *“Bathing my mother bothers me, but she is my mother. My culture does not allow bathing a parent, it is even worse if it is an opposite sex but for a girl it is somehow acceptable. In our case even if I were a boy, I would still do it.”*  *“Most challenging is cleaning and dressing the mzei. In our tradition, being a father, there are areas on the body that you shouldn’t see, but now because of the sickness, I have to keep the Mzei clean, you really have to do it but at the back of your mind, you feel oh no! traditionally am going beyond…”* |
| Nankinga et al.  (2020) | Dementia | *“I bring for him food to eat, bring him a jug of water to use for bathing and also collect his clothing for washing. I have to keep him safe and also remind him of something should he forget.”*  *“I help fasten buttons for him or if he wears a shirt inside-out, I help him wear it the right way.”*  *“I’m the one who cooks for him... I do his laundry, lay his bed and also cook for him food. Things like that.”*  *“I remind her when she forgets, when she misplaces something I tell her to take her time to search for it this helps her to find what she is looking for and even help her search for it or even at times I give her my own to stuff to use if she totally fails to get hers.”*  *“I also give her company by telling stories with her and also make sure she gets treatment when she is in need.”*  *“I always tell her how the drugs were prescribed to her. I remind her of the different schedules she is sup-posed to take them.”*  *“ she has her brother who usually comes to check on her. He usually brings her some beans when it’s the season or leaves her with some money to buy soap.”*  *“I see him become happy for example when he sends him money for treatment he comes back smiling and happy saying “my son has remembered me.”* |
| Nguyen et al. (2021) | Dementia | *“We work hard to take care of our parents, then our children will do the same to us. It comes around like that”*  *“I must do everything alone from A to Z. All the hard work, from start to end. You see, it is very hard being the eldest child; you must take care of your parents, you have your own work, and you have this task or the other task to complete too. Who else will do it for you? The brothers and sisters only come by every now and then.”*  *“I take care of bathing and washing laundry for him. He cannot do it himself. I carry him to the bath myself.”* |
| Ninnoni & Owoo (2023) | Prostate cancer | *“When we wake up in the morning, I clean him up; then I find out that he prefers breakfast. The truth is that he could not eat much, especially when the condition became serious. I do everything from cooking, washing and cleaning for him. if he needs to go to the toilet, I must assist him.”*  *“You can see that now he is weak, so I need to carry the water with the bucket to the bathroom for him; the primary care is eating/washing his clothing and ensuring that he takes his medications. Also, I take him on most hospital routines, especially for the reviews and investigations.”*  *“There is no toilet in the house. Hence, I must lift him into a chamber pot and back him to the wheelchair with no one to assist. At night he may also call to help him change positions or turn in bed.”*  *“If I were the one in his position now, he would be the one to care for me, and I know he will gladly do it.”*  *“He has taken care of our children; we live in our house with about ten extra rooms. So, he has tried for us; I cannot leave him now.”* |
| Nwakasi et al. (2023) | Cancer (breast) | *“During my therapy, I’d say I’m a very lucky person, I had my loved ones that encouraged me…sometimes I will be telling myself to not feel pity for myself since I had all these people that cared for me.”*  *“…my husband just told the boss, and he gave us N8 million ($19, 000) cash…the church knew I was going for treatment, and they supported, family supported.”*  *“My husband was supportive. In my own case after Jesus, it’s my husband. He is my second God. He was by my side from the beginning till now I’m talking to you. He goes to market for me up till now.”*  *“Even the time I wanted to commit suicide [because of the cancer], he [her husband] begged for the sake of the children that I should try to complete the treatment. We work together to gather money every three months. Sometimes we go together to the hospital for checkup, he really made the cancer treatment so easy for me.”*  *“My family members, which I just told you, that for now they are even tired, they don't even pick my calls anymore because of the challenges. Everywhere too there is no money, so I won't blame them.”*  *“ I know of cases [husbands abandoning wives] like that, that the husband said to her that she's a forgotten issue (she was in stage four), that no need for them to waste money. It is her daughter and friends that are helping her. I also know of another woman that got divorced by her husband because of her condition.”*  *“my very close friend never called for one day because she felt that cancer was my death sentence, they thought that I’m not going to make it.”* |
| Sadeghi-Mahalli et al. (2024) | Dementia (Alzheimer’s disease) | *“I used to live in a different city, but I moved here because of my father’s illness. Being closer has allowed me to help my mother more effectively. Now that I’m nearby, it’s much easier for me to assist her.”*  *“The main reason for what I do maybe is my feelings! Sense of duty! This feeling that I should be with my parents now. Because they were always with me and now it is my duty.”*  *“Once in the park, my husband became restless and unable to walk. A couple helped and took us home. They even came here a few days later and inquired about my husband’s condition. I was pleased.”* |
| Salifu et al. (2020) | Prostate cancer | *"We want to show him love and support by giving back what he did for us when we were young and fragile."*  *"I must be with him, especially this time he needs me the most, for better or worse, and in sickness and life. If I abandon him, I know I have offended the God I serve.*  *"I must care for him. God will not forgive me if we abandon him because he (dad) has done a lot for the family and me. I can’t even stand the criticisms from others. Everyone knows what my dad has done for us growing up."* |
| Shahrbabaki et al.  (2016) | Heart failure | *“When I see that my family love me despite my impatience and are open to accompanying me step-by-step, I get a sense of satisfaction and well-being.”*  *“When my wife is beside me, I feel reassured and calm. If my wife had not been there on the day I was diagnosed with heart failure, I could not have carried on.”*  *“My wife and children have the necessary knowledge about my disease and are sensitive to the timely use of my drugs. They have also encouraged me to quit smoking and take care of myself.”*  *“My hands and feet are swollen, and I cannot move. My son hugs me and moves me.”*  *“I am unable even to buy the drugs, but my children provide for my financial needs. I am really grateful to them”* |
| Sheikhpourkhani et al.  (2018) | Cancer (breast) | *“My sisters and my mother helped me a lot. Maybe that’s why I did not notice my ill-ness like others. They did what they could. These things raise my hope.”*  *“‘We used health insurance services. The charity institution paid the rest. Many times the costs were scanty and even free. All of these are hopes. At least among stresses due to illness and treatment, thinking about the cost of the disease is reduced.”* |
| Tsedze et al. (2025) | Cardiovascular disease | *“…I cannot do anything on my own without the support of my children and siblings…my feeding, going to the hospital for review, bathing, buying of medications, and other daily activities are the responsibility of my children and siblings. I could not have survived without them…this is not a condition (CVD) one could deal with all alone.”*  *“….my wife and children are the reason I am still alive. They provide me with all the needed support including going to church, making sure I take my medications, preparing my meals, and their words of encouragement alone are enough for me…they make me think less of the disease.”*  *“…as you can see, I am in a wheelchair and cannot do much for myself. Moreover, I am old and do not have the physical strength to deal with this condition (CVD). Thanks to my daughter for being there for me…she takes care of all my needs and constantly assures me that I will get better.”* |
| Widyastuti et al. (2023) | Dementia | “The primary family members consistently offer support, even if not all of them reside in Semarang. Those living outside the city provide financial assistance”  “If I need to be away from home for three days, my sister takes over the responsibility of caring for our mother”  “Now, my father’s physical health is stable because he regularly sees a doctor. I have a brother-in-law who assists in accompanying my father to medical appointments”  Every month, the children take turns visiting their father, which greatly motivates him |
| Zeilani et al. (2022) | Cancer | *“My family was there for me when I received my first course of chemo; I was feeling suffocated; I was not able to breathe because of my fears. But my family comforted me, they were as oxygen that helped me breathe.”*  *“My entire family were there for me, especially my sons and daughter, they never left me alone, they even asked their elder children to stay with me, I have never been left alone.”*  *“She (daughter) was there; she was available all of the time, especially when I got the bad news. I had cancer. She said, “Cancer cannot kill you; you are strong enough to defeat it, you have strong faith in Allah.” Then she started to perform ‘Doa’a’ asking Allah to save me.”*  *“I was badly shocked, they (doctors) decided to start chemotherapy, they asked me to take my hair off and wear a wig. I was out of my mind but when I asked my husband, he calmed me down and said that he loves me and will stay with my whatever it takes. His words are like a blossom to me.”*  *“I felt another life was granted to me after contacting with my children … They awakened me from coma, and I was with them all the time; they made my life full of joy … they wished for me to recover soon.”*  *“Once I was diagnosed with cancer, my husband started to care about me, helped me with housework, and take care of our children.”*  *All my sisters and brothers were around me and did not leave me alone. … My brother invited me to live with his home, as I was in need for help after the sessions of chemotherapy.”*  *“I am broke and do not have money to spend on my family; I asked my brother to lend me some. He gave me all the money that I needed.”*  *“My first challenge was to take care after my children. I have three kids. … thanks ‘Allah,’ my sister had the youngest one, and my brother took care of the other two. I felt relieved as they were in safe hands.”*  *“the chemo treatment started … I was frail and tired, that I could not go anywhere or do any work, my son left his job behind and stayed with me for 3 months.”*  *“the day of my diagnosis was the worst day of my life, my husband left me with three children and no money, later, I was divorced… I felt fed up with him.”* |
